# The miRNAs 203a/210‐3p/5001‐5p regulate the androgen/androgen receptor/YAP‐induced migration in prostate cancer cells

**DOI:** 10.1002/cam4.70106

**Published:** 2024-08-16

**Authors:** Chieh Huo, Ying‐Yu Kuo, Ching‐Yu Lin, Shine‐Gwo Shiah, Chia‐Yang Li, Shu‐Pin Huang, Jen‐Kun Chen, Wen‐Ching Wang, Hsing‐Jien Kung, Chih‐Pin Chuu

**Affiliations:** ^1^ Institute of Cellular and System Medicine National Health Research Institutes Zhunan Taiwan; ^2^ Ph.D. Program for Cancer Molecular Biology and Drug Discovery Taipei Medical University Taipei Taiwan; ^3^ National Institute of Cancer Research National Health Research Institutes Zhunan Taiwan; ^4^ Graduate Institute of Medicine, College of Medicine Kaohsiung Medical University Kaohsiung Taiwan; ^5^ Department of Urology, School of Medicine, College of Medicine Kaohsiung Medical University Kaohsiung Taiwan; ^6^ Institute of Biomedical Engineering and Nanomedicine National Health Research Institutes Zhunan Taiwan; ^7^ Institute of Molecular and Cellular Biology National Tsing Hua University Hsinchu Taiwan; ^8^ Ph.D. Program for Aging China Medical University Taichung Taiwan; ^9^ Biotechnology Center National Chung Hsing University Taichung Taiwan; ^10^ Department of Life Sciences National Central University Taoyuan Taiwan

**Keywords:** androgen, AR, miRNA, prostate cancer metastasis, YAP

## Abstract

**Background:**

Prostate cancer (PCa) patients with elevated level of androgen receptor (AR) correlate with higher metastatic incidence. Protein expression of AR and its target gene prostate‐specific antigen (PSA) are elevated in metastatic prostate tumors as compared to organ‐confined tumors. Androgen treatment or elevation of AR promotes metastasis of PCa in cell culture and murine model. However, under androgen depleted condition, AR suppressed cell mobility and invasiveness of PCa cells. Androgen deprivation therapy in PCa patients is associated with higher risk of cancer metastasis. We therefore investigated the dual roles of AR and miRNAs on PCa metastasis.

**Methods:**

The PC‐3^AR^ (PC‐3 cells re‐expressing AR) and LNCaP cells were used as PCa cell model. Transwell migration and invasion assay, wound‐healing assay, zebrafish xenotransplantation assay, and zebrafish vascular exit assay were used to investigate the role of AR and androgen on PCa metastasis. Micro‐Western Array, co‐immunoprecipitation and Immunofluorescence were applied to dissect the molecular mechanism lying underneath. The miRNA array, miRNA inhibitors or plasmid, and chromatin immunoprecipitation assay were used to study the role of miRNAs on PCa metastasis.

**Results:**

In the absence of androgen, AR repressed the migration and invasion of PCa cells. When androgen was present, AR stimulated the migration and invasion of PCa cells both in vitro and in zebrafish xenotransplantation model. Androgen increased phospho‐AR Ser81 and yes‐associated protein 1 (YAP), decreased phospho‐YAP Ser217, and altered epithelial‐mesenchymal transition (EMT) proteins in PCa cells. Co‐IP assay demonstrated that androgen augmented the interaction between YAP and AR in nucleus. Knockdown of YAP or treatment with YAP inhibitor abolished the androgen‐induced migration and invasion of PCa cells, while overexpression of YAP showed opposite effects. The miRNA array revealed that androgen decreased hsa‐miR‐5001‐5p but increased hsa‐miR‐203a and hsa‐miR‐210‐3p in PC‐3AR cells but not PC‐3 cells. Treatment with inhibitors targeting hsa‐miR‐203a/hsa‐miR‐210‐3p, or overexpression of hsa‐miR‐5001‐5p decreased YAP expression as well as suppressed the androgen‐induced migration and invasion of PCa cells. Chromatin immunoprecipitation (ChIP) assay demonstrated that AR binds with promoter region of has‐miR‐210‐3p in the presence of androgen.

**Conclusions:**

Our observations indicated that miRNAs 203a/210‐3p/5001‐5p regulate the androgen/AR/YAP‐induced PCa metastasis.

## Graphical abstract

Our finding provided new knowledge for understanding how androgen determines the dual roles of AR on prostate cancer metastasis. We made new discovery that androgen promotes metastasis of AR‐positive PCa via regulation of hsa‐miR‐203a, hsa‐miR‐210‐3p, hsa‐miR‐5001‐5p, and YAP. The finding of the pathway AR/androgen/miRNAs/YAP offered an opportunity for developing novel drugs to delay or to suppress the PCa metastasis.

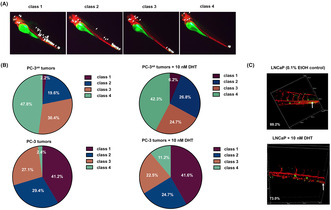

## 1 BACKGROUND

Previous studies suggested a stimulating role of androgen receptor (AR) on prostate cancer (PCa) metastasis. Protein expression of AR and its target gene prostate‐specific antigen (PSA) was elevated in prostate tumors from PCa patients with metastasis as compared to patient with organ‐confined tumors.^1^, ^2^, ^3^ Prostate tumors with higher AR expression level correlated with worse clinical outcome.^3^ Elevation of AR promoted metastasis of PCa by induction of epithelial–mesenchymal transition (EMT), activation of elongation factor eIF5A2, and reduction of KAT5.^3^, ^4^ Knockdown of AR decreased migration and invasion of PCa cells.^3^ Androgen enhanced metastasis of prostate tumors in murine PCa model via activation of TMPRSS2/HGF/c‐Met signaling axis,^5^ induction of matriptase, and increase of extracellular matrix degradation.^6^ Treating AR‐positive PCa cell lines with androgens resulted in increased uptake of fatty acids, cholesterol, and low‐density lipoprotein particles, which were associated with disease progression and metastasis of PCa.^7^ However, other studies suggested a suppressive role of AR on PCa metastasis. Under androgen‐depleted condition, AR suppressed cell mobility and invasiveness of PCa cells via inhibition of EMT regulatory proteins.^8^ PCa cells were found to recruit pre‐adipocytes, which promoted invasion of PCa cells via suppression of AR, inhibition of miR‐301a, and activation of TGF‐β1/Smad/MMP9 signaling.^9^ Androgen deprivation therapy (ADT) for months was reported to increase expression of N‐cadherin in prostate tumors, which was associated with cancer metastasis.^10^ ADT in xenograft model increased bone metastasis of prostate tumors via repression of transcription factor SPDEF and induction of TGFB I.^11^ These contradictory findings suggested that AR may have dual roles on PCa metastasis and the molecular mechanism lying underneath may be complicated.

MicroRNAs (miRNA or miR) are non‐coding short RNAs that regulate gene expression. The miRNAs have been found to regulate PCa metastasis and AR signaling in PCa cells. The miR‐9, miR‐21, miR‐181a, and miR‐186 have been reported to promote EMT, cancer metastasis and cell proliferation in PCa cells, while miR‐33a‐5p, miR‐34, miR132, miR‐212, miR‐145, miR‐141‐3p, miR‐200, miR‐204‐5pmiR‐532‐3p, miR‐335, miR‐543, miT‐505‐3P, miR‐19a‐3p, miR‐802, miR‐940, and miR‐3622a have been shown to suppress EMT and cancer metastasis in PCa cells.^12^ Additionally, miR‐205, miR30b, miR‐30d, miR‐31, miR‐124a, miR‐320, and miR‐212 have been reported to inhibit AR expression.^13^ As miRNAs may play important role in PCa metastasis and AR signaling, we examined if regulation of migration and invasion of PCa cells by AR and androgen is regulated by miRNAs.

## 2 MATERIALS AND METHODS

### 2.1 Cell culture, chemicals, and plasmids

PC‐3 and LNCaP FGC cells were purchased from Bioresource Collection and Research Center (Hsinchu City, Taiwan). PC‐3 cells were transfected with LNCX‐2‐wild type AR plasmid with neomycin G418 selection to generate PC‐3^AR^.^14^ Cells were maintained in DMEM (Gibco/Invitrogen, Waltham, MA, USA) supplemented with 10% charcoal‐stripped fetal bovine serum (CS‐FBS).^15^ FBS and dihydrotestosterone (DHT) was from Biological Industries (Beit Haemek, Israel) and Sigma Aldrich (St. Louis, MO, USA), respectively.

### 2.2 Zebrafish xenotransplantation assay

Experimental procedures on fish embryos were approved by NHRI's Institutional Animal Care and Use Committee (NHRI‐IACUC‐109036‐A). Experimental procedures are explained in Supplemental Methods. Photos of embryos bearing tumor cells was taken by a fluorescent microscope (ZEISS Discovery V8, 20X magnification). The fluorescent area of CFSE signal were quantified using MetaXpress (Molecular Devices, San Jose, CA, USA). The transverse length of fluorescent signal was used to measure the metastatic distance and the metastatic ability was clustered into Class 1 (cells with fluorescence dye appear in head, body, and tail blood vessels), Class 2 (cells with fluorescence dye appear in head and tail blood vessels as well as a small portion in the body), Class 3 (cells with fluorescence dye appear only in the head and tail blood vessels), and Class 4 (the amount of fluorescent cells is very little amount or almost undetectable).

### 2.3 Zebrafish vascular exit assay

Three days after injection of fluorescent‐labeled cancer cells, a microscope (ZEISS Discovery V8, magnification 80×) was used to examine if fluorescent cancer cells were located outside of blood vessel. If one or more fluorescent cancer cells were observed, the blood vessel area will be considered as positive for cancer metastasis and will further be confirmed with confocal microscope (Nikon A1R, magnification 200×).

### 2.4 RNA interference studies

YAP and AR were knocked down with Dharmacon ON‐TARGETplus SMART pool siRNA probes (Dharmacon, Lafayette, CO, USA), and non‐targeting control pool was as a control. The siRNA was transfected at a concentration of 40 nM with lipofectamine RNAiMAX reagent (Invitrogen).

### 2.5 Immunoblot analysis

Western blotting was conducted as previously described.^16^ Information of antibodies detecting the specific proteins are listed as following: androgen receptor (AR) and c‐Myc were from Abcam (Cambridge, MA, USA); CDK1, CDK5, CDK9, β‐catenin, c‐Jun, GSK‐3α, Snail, Slug, TAZ, vimentin, and YAP were from Cell signaling (Danvers, MA, USA); GAPDH was from Novus (Littleton, CO, USA); E‐cadherin and N‐cadherin were from BD (Franklin Lakes, NJ, USA); Twist1 was from Genetex (Irvine, CA, USA); phospho‐AR S81 and IgG were from Santa Cruz (Dallas, TX, USA). All experiments were repeated at least three times.

### 2.6 Immunofluorescence

Cells were seeded in Millicell EZ slide (Merck Millipore, Burlington, MA, USA) and treated with DHT or not for 48 h. Cells were fixed with 4% para‐formaldehyde on ice for 15 min and permeabilized in 0.3% Triton X‐100 (in PBS) for 10 min. Samples were then blocked for an hour and were stained with indicated antibody for 16 h at 4°C. Alexa Fluor 488 dye and Alexa Fluor 594 dye (Thermo Fisher Scientific) were used as secondary antibody for green and red fluorescent dye. The cell nuclei were stained with DAPI.

### 2.7 Wound healing assay

Cells which pre‐treated with 10 nM DHT or control vehicle for 48 h and cells were then seeded at 4 × 10 cells/100 μL into ibidi culture inserts in a 24‐well plate. After 24 h, wound healing assay was performed with ibidi culture insert (Applied Biophysics, Troy, NY, USA). Cells were monitored with live imaging image system (Leica AF 6000 LX, Leica, Wetzlar, Germany).

### 2.8 Transwell cell migration and invasion assay

Migration and invasion assays were performed with a transwell kit from BD Bioscience as previously described.^17^

### 2.9 Co‐immunoprecipitation

Detail of Co‐immunoprecipitation is provided in the Supplemental Materials & Methods.

### 2.10 Micro‐western arrays (MWA)

The MWA was conducted as previously described.^18^ Odyssey Infrared Imaging System and Odyssey 3.0 software were used for blot imaging scan and quantification, respectively. Antibodies used in present study are reported in Table S1.

### 2.11 The miRNA array

We used 1000 ng of each sample to join a tail of Poly(A) with a PolyA polymerase and labeled the samples with biotin following the protocol of FlashTag Biotin HSR RNA Labeling Kit for Applied Biosystems GeneChip miRNA arrays (Genisphere). 21.5 μL of Biotin‐labeled sample were hybridized for 16 h at 48°C on GeneChip miRNA 4.0 Array. GeneChips were washed and stained in the Affymetrix Fluidics Station 450. GeneChips were scanned using the Affymetrix GeneArray Scanner 3000 7G. Heatmap was generated by Partek Genomics Suite.

### 2.12 The miRNA extraction and qPCR analysis

Detail of micro‐RNA extraction is provided in the Supplemental Materials & Methods. The sequences for qPCR primers were as follows:

hsa‐miR‐26a‐5p TTC AAG TAA TCC AGG ATA GGC

hsa‐miR‐203a GTG AAA TGT TTA GGA CCA CTA G

hsa‐miR‐210‐3p CTG TGC GTG TGA CAG CGG CTG A

hsa‐miR‐509‐3p TGA TTG GTA CGT CTG TGG GTA G

hsa‐miR‐532‐3p CCT CCC ACA CCC AAG GCT TGC A

hsa‐miR‐5001‐5p AGG GCT GGA CTC AGC GGC GGA GCT

### 2.13 Real‐time quantitative PCR

RNA was extracted with a RNeasy Mini Kit (Qiagen, Germantown, MD, USA). The primer sequences were as follows: YAP forward: 5′‐GGTGCCACTGTTAAGGAAAGG‐3′ and reverse: 5′‐GTGAGGCCACAGGAGTTAGC‐3′; CTGF forward: 5′‐TGGTGCAGCCAGAAAGCTC‐3′ and reverse: 5′‐CCAATGACAACGCCTCCTG‐3′; CYR61 forward: 5′‐TTCTTTCACAAGGCGGCACTC‐3′ and reverse: 5′‐AGCCTCGCATCCTATACAACC‐3′. Expression of GAPDH gene was used as loading control.

### 2.14 Transfection of miRNA

20 nM of miR‐203a‐3p inhibitor sequence (IH‐300562‐05, Dharmacon, Lafayette, USA) or miR‐210‐3p inhibitor sequence (IH‐300565‐05, Dharmacon, Lafayette, USA) was transfected into cells using Lipofectamine RNAiMAX transfection reagent (Thermo Fisher). 2 μg of plasmid of miR‐5001 (SC401955, OriGene, Rockville, USA) were transfected into cells in 6‐well using PolyJet reagent (SignaGen Laboratories, Frederick, MD, USA). Sixteen hours post‐transfection, medium was replaced with fresh culture medium. After 48 h for recovery, cells were harvested for experiments.

### 2.15 Chromatin immunoprecipitation (ChIP) assay

ChIP assays were performed by using a Magna ChIP A/G assay kit (Millipore, Burlington, MA, USA). Briefly, 2 × 10^7^ PC‐3 AR cells were cross‐linked with 1% fresh formaldehyde for 10 min and add glycine to a final concentration of 125 mM for 5 min at RT. The cell lysates were sonicated with Covaris‐M220 for cell chromatin shearing and subjected to IP using mouse anti‐androgen receptor antibody (Santa Cruz, TX, USA), mouse IgG (Bethyl) antibodies. The precipitated DNAs were analyzed and quantified by using SYBR Green/ROX real‐time PCR (Thermo Fisher Scientific, Waltham, MA, USA). A quantitative GAPDH control primer was provided from Magna ChIP kit; the promoter primer sequences were as follows: miR210‐3p Forward: 5′‐AGGGGAGGACAAAGAGCA‐3′, miR210‐3p Reverse: 5′‐TTCAGACGTGGAAGTATCGGA −3′.

### 2.16 Statistical analysis

Statistical analyses were performed using Student's *t*‐test and one‐way ANOVA. Error bars represented standard error. Statistically significant *p* values are abbreviated as follows: *, *p* < 0.05; **, *p* < 0.01; ***, *p* < 0.001.

## 3 RESULTS

### 3.1 Activated androgen receptor promotes the migration and invasion of AR‐positive PCa cells

**FIGURE 1:**
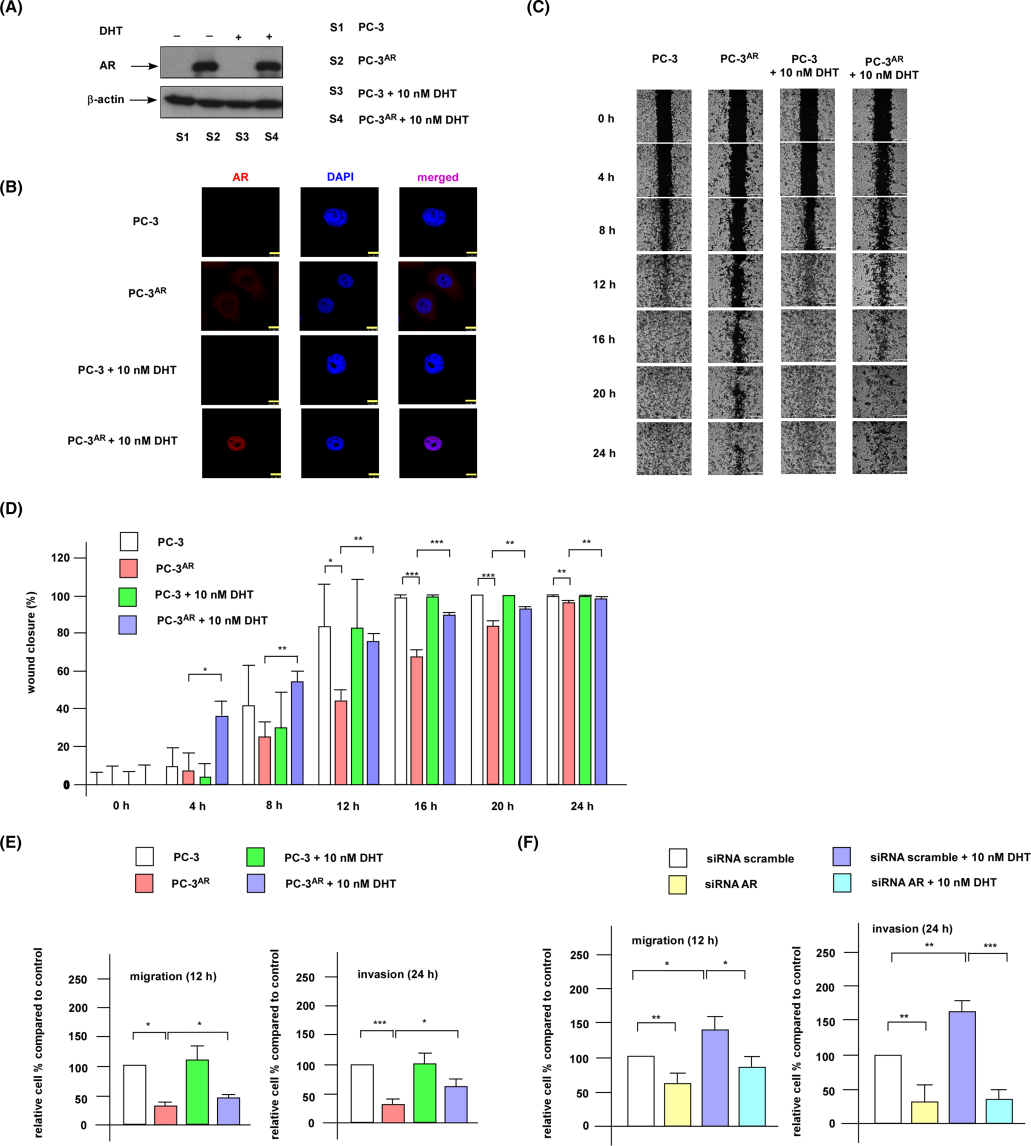
Androgen promotes migration and invasion of PC‐3^AR^ cells. (A) Protein expression of AR in PC‐3 cells and PC‐3^AR^ cells with or without androgen (10 nM dihydrotestosterone (DHT)) treatment. (B) Expression and location of AR in control PC‐3 cells and PC‐3^AR^ cells was examined by immunofluorescence staining using Leica TCS SP5 AOBS Confocal Spectral Microscopy with red and blue fluorescence for AR and DAPI staining, respectively. A yellow scale bar showing 7.5 μM was arranged at the lower right part of images. (C) Wound healing assay was performed to determine the effect of androgen and AR on the migration of PC‐3 and PC‐3^AR^ cells. (D) Quantification of wound healing assay in (C) was shown. (E) Cell migration and invasion of PC‐3 and PC‐3^AR^ cells with or without treatment of 10 nM DHT were determined by a transwell chamber assay. (F) Cell migration and invasion of LNCaP C4‐2B siRNA scramble control and LNCaP C4‐2B AR siRNA knockdown cells with or without treatment of 10 nM DHT were determined by a transwell chamber assay. Asterisks *, **, and *** represent statistically significant difference *p* < 0.05, *p* < 0.01, and *p* < 0.001, respectively.

To explore the effects of androgen receptor (AR) and androgen on PCa metastasis, we compared the effects of androgen on migration and invasion of AR‐negative PC‐3 and AR‐positive PC‐3^AR^ cells. PC‐3^AR^ is a cell line we previously established by stably re‐expressing full length human AR in PC‐3 cells^8^ (Figure 1A). AR was undetectable in PC‐3 cells (Figure 1B). In PC‐3^AR^ cells, AR located in the cytoplasm when androgen was absent but translocated into the nucleus when androgen was present (Figure 1B). Migration and invasion of PC‐3 cells were not affected by androgen (Figure 1C–E). However, the cell mobility and invasiveness of PC‐3^AR^ cells was slower than PC‐3 when androgen was absent. When androgen was present, androgen increased the cell mobility and invasiveness of PC‐3^AR^ cells (Figure 1C–E). Androgen also enhanced the migration and invasion of LNCaP C4‐2B cells (Figure 1F). Knockdown of AR with siRNA in LNCaP C4‐2B cells reduced the migration and invasion of LNCaP cells as well as hindered the androgenic stimulation on migration and invasion (Figure 1F).

**FIGURE 2:**
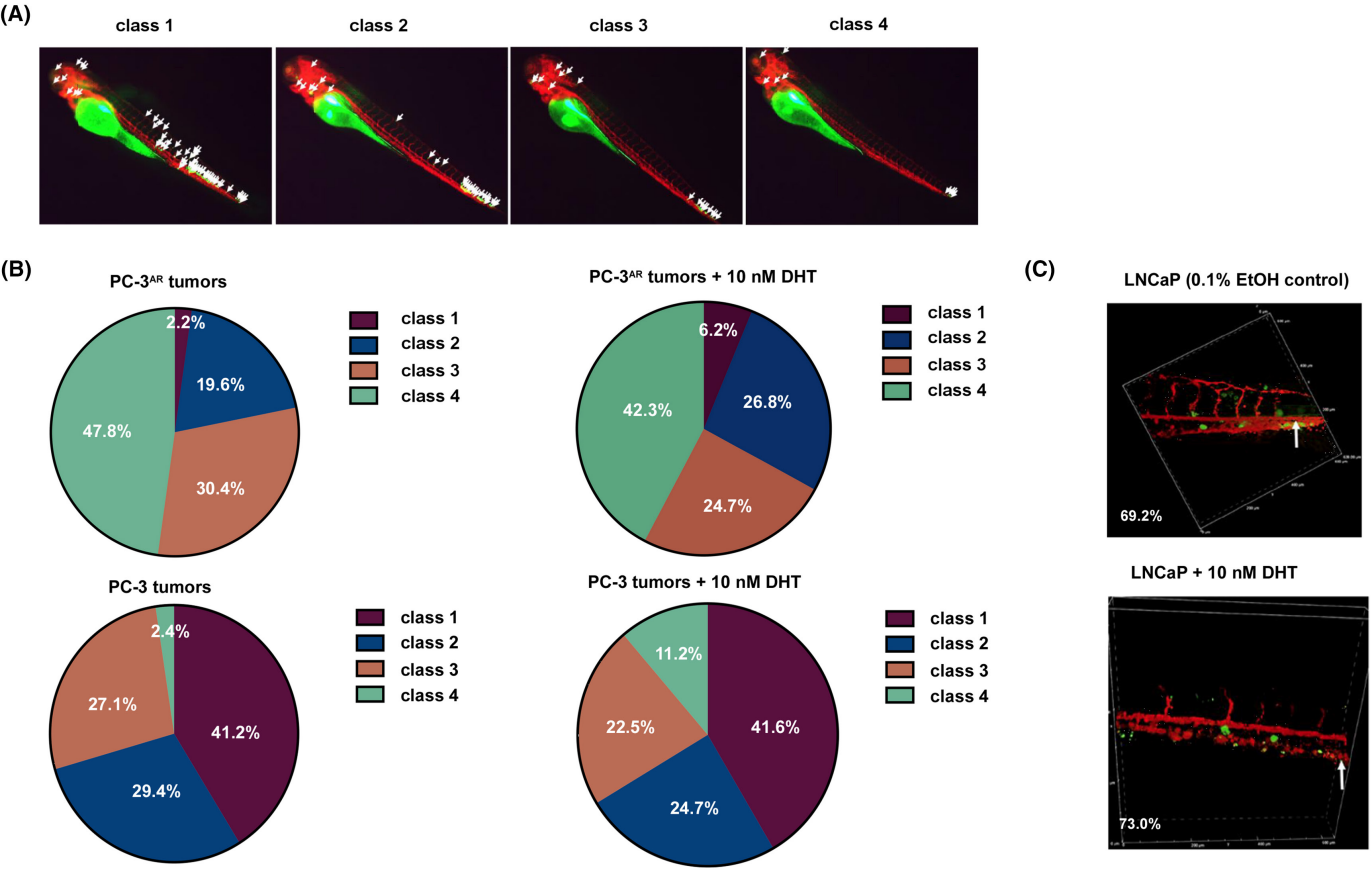
Zebrafish xenotransplantation model analysis of effects of androgen treatment on metastasis of PC‐3^AR^ tumors. (A) Representative images of Class 1, Class 2, Class 3, and Class 4 are shown. (B) A quantification of the percentage of Class 1‐Class 4 in zebrafish carrying PC‐3^AR^ or control PC‐3 tumors incubated with control vehicle (0.1% ethanol) versus zebrafish carrying PC‐3^AR^ tumors incubated with 10 nM dihydrotestosterone (DHT). (C) Migrated fluorescent LNCaP cells across blood vessels in zebrafish treated with control vehicle (0.1% ethanol) or 10 nM DHT were assayed by vascular exit assay and quantified with counting using microscope.

To determine if androgen stimulates PCa metastasis *in vivo*, we performed zebrafish xenotransplantation experiment. The transverse length of fluorescent signal was measured to monitor the metastasis of fluorescent human PCa cells in zebrafish. The fluorescent human PCa cells in fish were clustered from highest migratory ability (Class 1) to lowest migratory ability (Class 4) (Figure 2A). Androgen significantly increased metastasis of PC‐3^AR^ cells, as Class 1 PC‐3^AR^ cells increased from 2.2% to 6.2% and Class 2 PC‐3^AR^ cancer cell increased from 19.6% to 26.8% in fish cultured with androgen as compared to those in fish cultured without androgen. On the other hand, androgen had little effect on migration of PC‐3 cells which were AR‐negative. Under androgen treatment, Class I PC‐3 cells changed from 41.2% to 41.6%, and Class II PC‐3 cells changed from 29.4% to 24.7% (Figure 2B). A vascular exit assay revealed that androgen treatment also increased the migrated LNCaP cells in zebrafish (Figure 2C). These observations suggested that androgen promoted the cell mobility and invasiveness of AR‐positive PCa cells in vivo. Control PC‐3 cells and PC‐3^AR^ cells were injected into castrated or intact nude mice to examine the effect of androgen ablation and re‐expression of AR on metastasis and angiogenesis of prostate xenografts. Re‐expression of AR reduced angiogenesis and metastatic tumors of PC‐3 cells (Figure S1). We did not observe a difference in number of metastatic PC‐3 tumors and PC‐3^AR^ tumors between intact and castrated nude mice, possibly due to the relatively small number of metastatic tumors developed in the nude mice.

### 3.2 Androgen promotes EMT and YAP signaling in PC‐3^AR^ cells

**FIGURE 3:**
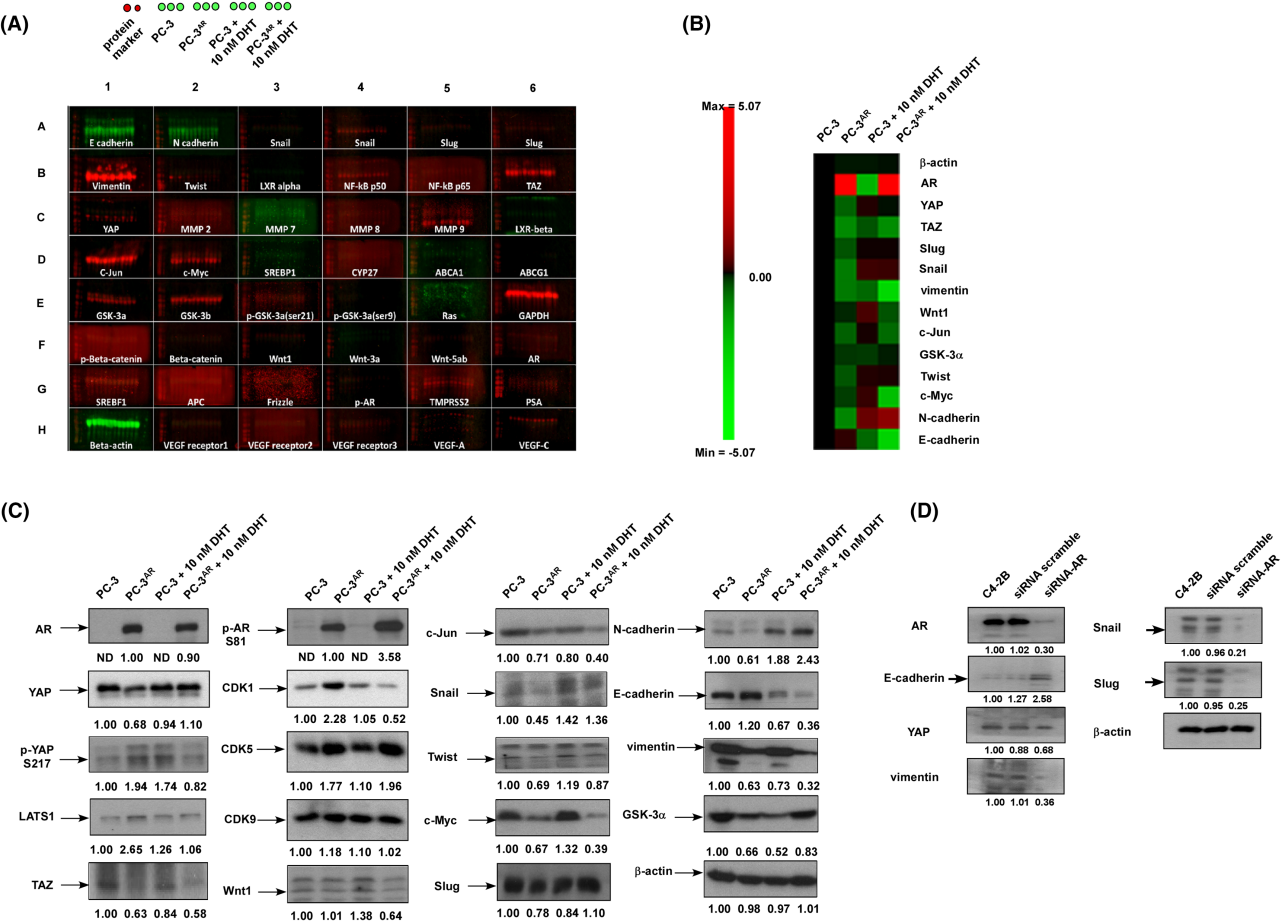
Micro‐western array (MWA) analysis of protein expression profile of PC‐3 and PC‐3^AR^ cells with or without androgen treatment. (A) A representative image of MWA assay of PC‐3 and PC‐3^AR^ cells with or without 10 nM dihydrotestosterone (DHT) treatment for 48 h. Red and green color was for anti‐rabbit and anti‐mouse 2nd antibody, respectively. The 12 samples printed in each well from left to right were triplicates of PC‐3 cells without DHT, PC‐3^AR^ cells without DHT, PC‐3 cells being treated with 10 nM DHT, and PC‐3^AR^ cells being treated with 10 nM DHT. (B) Proteins of which the expression level was significantly changed for at least 2‐fold was shown. The heatmap demonstrated the log_2_ value of the changes of protein abundance. (C) Expression of AR, phospho‐AR Ser81, YAP, phospho‐YAP Ser217, CDK1, CDK5, CDK9, LATS1, TAZ, Wnt1, c‐Jun, Snail, Twist, c‐Myc, Slug, N‐cadherin, E‐cadherin, vimentin, and GSK‐3α proteins in PC‐3 and PC‐3^AR^ cells being treated with or without 10 nM DHT was assayed by western blotting. The β‐actin was used as loading control. (D) Expression of AR, E‐cadherin, YAP, vimentin, Snail, and Slug proteins in LNCaP C4‐2B cells being treated with or without 10 nM DHT was assayed by western blotting. The β‐actin was used as loading control. All experiments were repeated at least three times.

Micro‐Western Array (MWA) was applied to determine the mechanism how androgen enhances the motility and invasiveness of PCa cells (Figure 3A). Under androgen‐depleted condition, AR suppressed the Yes‐associated protein 1 (YAP), TAZ, Snail, Slug, Wnt1, vimentin, GSK3α, c‐Jun, c‐Myc, Twist, and N‐cadherin proteins but increased protein abundance of E‐cadherin (Figure 3B) in PC‐3^AR^ cells as compared to PC‐3 cells. When androgen was present, AR increased the protein abundance of YAP, Slug, Snail, Twist, GSK3α, and N‐cadherin but decreased the protein level of E‐cadherin, c‐Myc, and vimentin in PC‐3^AR^ cells as compared to PC‐3 cells (Figure 3B). Similar results were confirmed by conventional western blot. In the absence of androgen, AR decreased YAP, TAZ, Snail, Slug, c‐Myc, c‐Jun, GSK‐3α, Twist, N‐cadherin, and vimentin while increased the expression of E‐cadherin, CDK1, CDK5, phospho‐YAP S127, phospho‐AR S81, and LATS1 proteins in PC‐3^AR^ cells as compared to PC‐3 cells (Figure 3C). Androgen increased the level of YAP, Slug, Snail, GSK‐3α, Twist, CDK5, phospho‐AR S81, and N‐cadherin proteins but decreased the protein abundance of E‐cadherin, CDK1, phospho‐YAP S127, and LATS1 in PC‐3^AR^ cells (Figure 3C). In LNCaP cells, siRNA knockdown of AR increased E‐cadherin but decreased YAP, vimentin, Snail, and Slug (Figure 3D).

### 3.3 AR interacts with YAP in the presence of androgen

**FIGURE 4:**
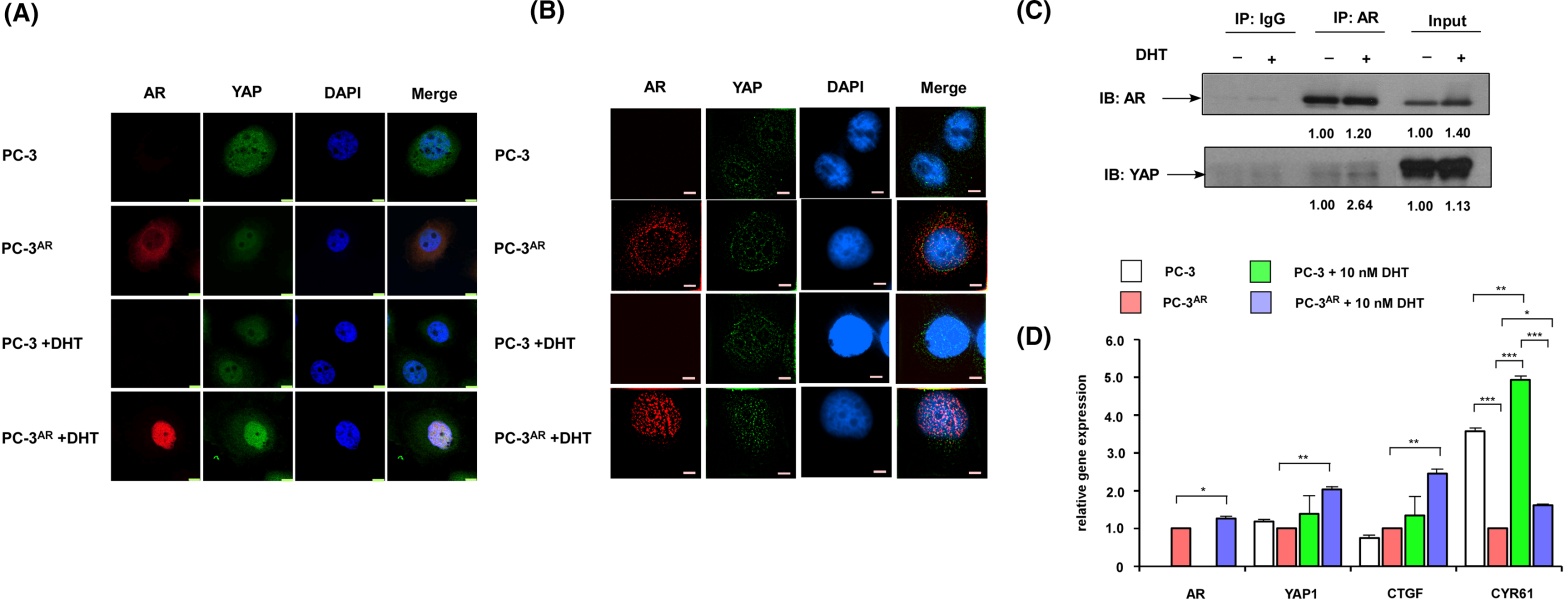
Immunofluorescence analysis of the co‐localization of AR and YAP in nucleus of PCa cells with or without androgen treatment. Localization of AR and YAP in control PC‐3 and PC‐3^AR^ cells being treated with or without 10 nM dihydrotestosterone (DHT) for 48 h was observed by confocal microscopy (A) and Nikon's Structured Illumination microscope imaging (N‐SIM) (B). The column from left to right shows AR (red), YAP (green), DAPI (blue) and merged images. The magnification was 63× and 60×, respectively. The scale bars in (A) (bright yellow) and (B) (white) represent 75 and 5 μm, respectively. (C) Co‐immunoprecipitation analysis of the interaction between AR and YAP in PC‐3^AR^ cells in the presence or absence of 10 nM DHT for 48 h. IgG was used as a negative control. (D) Gene expression of AR, YAP1, CTGF, and CYR61 in PC‐3 and PC‐3^AR^ cells being treated with or without 10 nM DHT for 48 h was analyzed with qRT‐PCR. GAPDH was used as loading control.

When the Hippo pathway is activated, YAP is phosphorylated at both serine 127 and serine 381.^19^ Phosphorylation at Ser127 recruits 14‐3‐3 proteins to bind YAP and retains YAP in cytoplasm. YAP is therefore suppressed by spatial separation from its target transcription factors which located in nucleus.^19^ We observed that androgen increased expression of YAP but decreased phosphorylation of Ser127 on YAP (Figure 3C). We next examined if AR directly interacts with YAP when androgen is present. Confocal microscopy revealed that YAP co‐localized with AR in nucleus when androgen was present (Figure 4A). Nikon's Structured Illumination microscope imaging (N‐SIM), which has better spatial resolution than confocal microscope, clearly demonstrated that AR co‐localized with YAP in nucleus only when androgen was present (Figure 4B). Co‐immunoprecipitation (co‐IP) revealed that androgen augmented the interaction between AR and YAP (Figure 4C). Additionally, androgen elevated the gene expression of YAP1, connective tissue growth factor (CTGF) and cysteine‐rich angiogenic inducer 61 (CYR61) in PC‐3^AR^ cells (Figure 4D). CTGF and CYR61 are target genes of YAP. These findings indicated that androgen is required for the interaction and activation of AR‐YAP complex.

**FIGURE 5:**
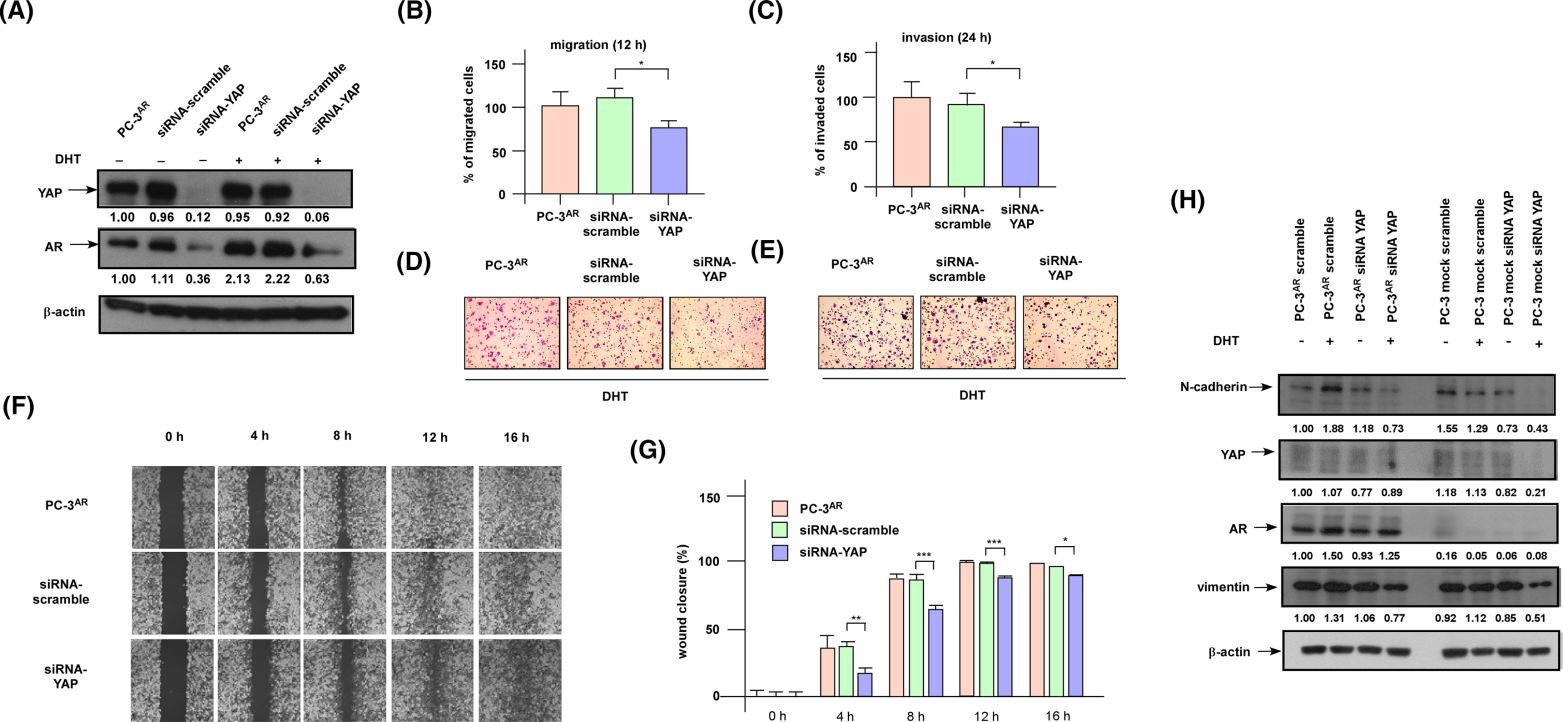
Knockdown of YAP in PC‐3^AR^ cells hindered androgen‐promoted cell migration. (A) Protein expression in PCa cells with YAP siRNA or scramble control with or without androgen treatment (10 nM dihydrotestosterone (DHT), 48 h) was determined by western blotting assay. Migration (B) and invasion (C) of PCa cells with YAP siRNA or scramble control, all treated with androgen (10 nM DHT, 48 h), were determined by transwell migration and invasion assays, respectively. Images of migration and invasion transwell assays were shown in (D) and (E), respectively. Migration of PCa cells with YAP siRNA or scramble control, treated with 10 nM DHT for 0, 4, 8, 12, 16 h, was examined with wound healing assay (F). The magnification of the microscope is 100×. The wound closure area was measured and quantified in (G). Protein expression of N‐cadherin, YAP, AR, and vimentin in PC‐3^AR^ and PC‐3 mock cells being with or without siRNA knockdown of YAP in the presence or absence of androgen was examined with western blotting assay. The β‐actin was used as loading control. (H) Effect of YAP knockdown on protein expression of N‐cadherin, vimentin, AR and YAP in PC‐3^AR^ and PC‐3 cells.

**FIGURE 6:**
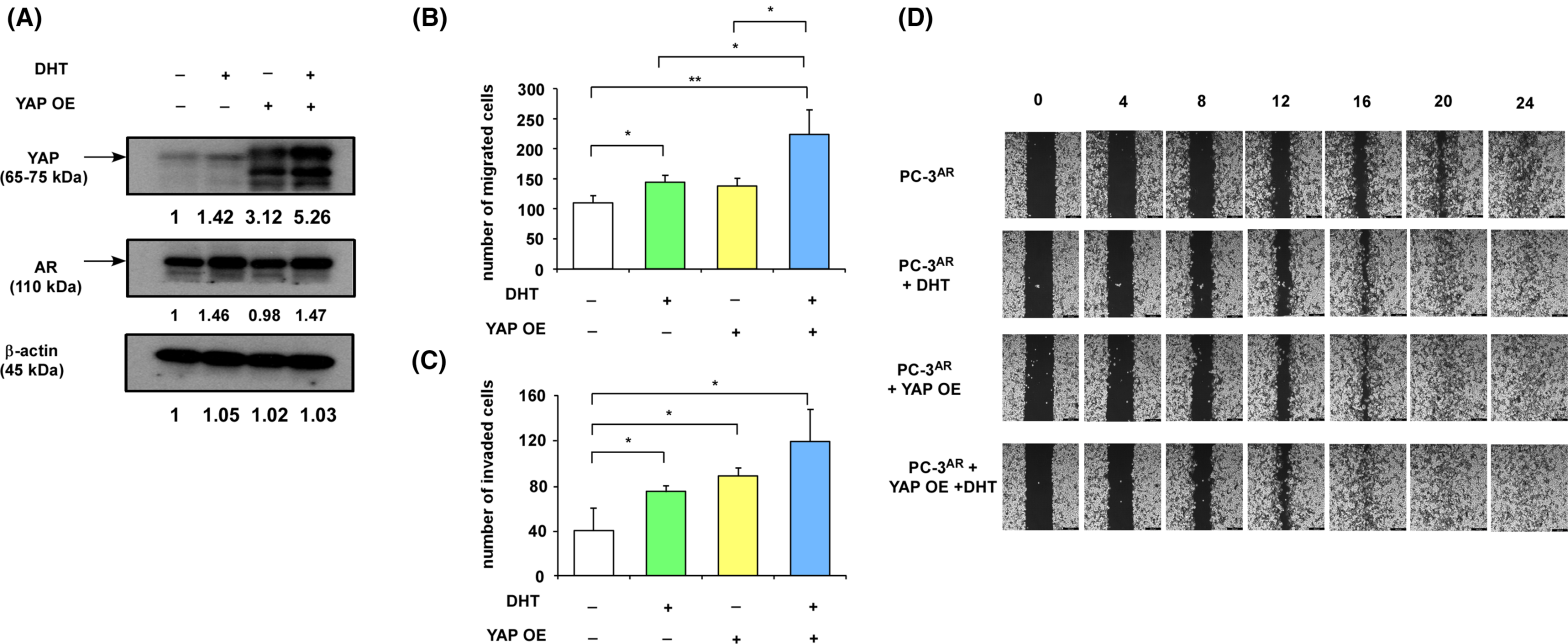
Overexpression of YAP promotes migration and invasion of PCa cells in the presence of androgen. (A) Protein expression of YAP and AR in PC‐3^AR^ cells with or without 10 nM dihydrotestosterone (DHT) for 48 h or YAP overexpression was determined by western blotting assay. Migration (B) and invasion (C) of the above PCa cells were determined by a transwell assay. Cells were seeded at identical number and counted after 12 h for a cell migration assay and 24 h for an invasion assay, respectively. Asterisks * and ** represent statistically significant difference *p* < 0.05 and *p* < 0.01, respectively. (D) Wound healing assay was performed to determine the effect of androgen treatment (10 nM DHT, 48 h) or YAP overexpression on the migration ability of PC‐3 cells 0–24 h after cell seeding. The magnification of the Leica AF 6000 LX microscope is 100×.

To determine the role of YAP on migration and invasion of PC‐3^AR^ cells, we knocked down the YAP in PC‐3^AR^ cells (Figure 5A). Interestingly, knockdown of YAP significantly reduced protein level of AR in PC‐3^AR^ cells (Figure 5A). Knockdown of YAP compromised the effects of androgen and reduced the androgen‐induced migration and invasion (Figure 5B–E). A wound healing assay revealed that YAP knockdown reduced the androgen‐induced mobility of PC‐3^AR^ cells (Figure 5F,G). Knockdown of YAP hindered the androgenic stimulation of N‐cadherin, AR, and vimentin in PC‐3^AR^ cells (Figure 5H). On the other hand, overexpression of YAP (Figure 6A) showed opposite effects. In androgen‐depleted condition, overexpression of YAP slightly increased the migration and invasion of PC‐3^AR^ cells (Figure 6B–D). In the presence of androgen, overexpression of YAP further enhanced the migration, invasion, and mobility (Figure 6B–D) of PC‐3^AR^ cells.

**FIGURE 7:**
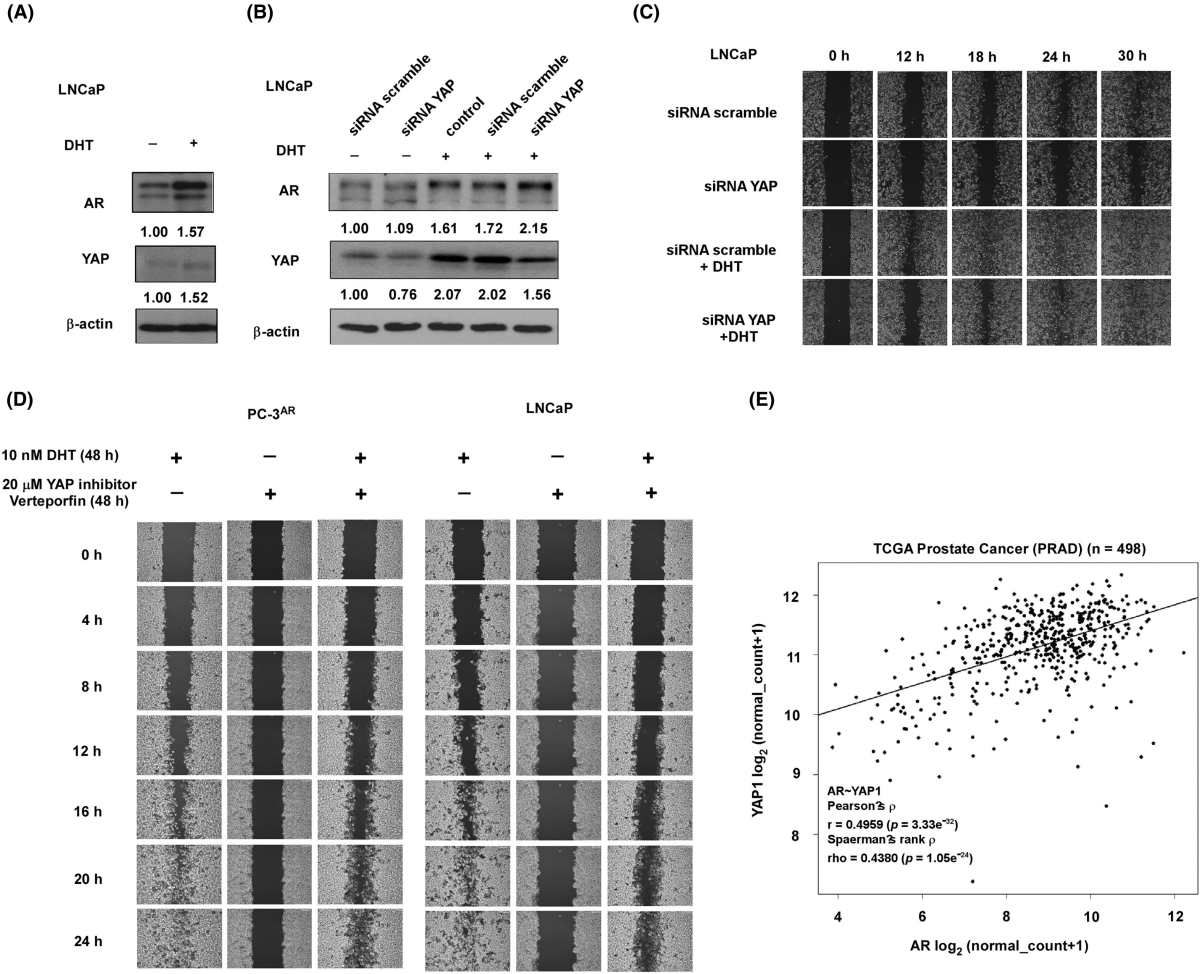
Knockdown of YAP or treatment of YAP inhibitor hindered androgen‐promoted cell migration in LNCaP and PC‐3^AR^. Protein expression of AR and YAP in LNCaP cells with or without of 10 nM dihydrotestosterone (DHT) (48 h) (A) or in LNCaP cells transfected with YAP siRNA or scramble control (B) in the presence or absence of 10 nM DHT (48 h) was determined. (C) LNCaP cells with scramble control or YAP siRNA were treated with 10 nM DHT for 0, 12, 18, 24, 30 h, and migration of these cells was examined with wound healing assay. The magnification of the microscope is 100×. (D) Migration of PC‐3^AR^ or LNCaP cells being treated with 48 h of 10 nM DHT and/or 20 μM YAP inhibitor Verteporfin for 0, 4, 8, 12, 16, 20, 24 h was examined with wound healing assay. The magnification of the microscope is 100×. (E) Correlation between the gene expression level of AR and YAP1 in TCGA prostate cancer database (*n* = 498) was analyzed. The gene expression level was demonstrated using log_2_ value.

Androgen increased the expression of both AR and YAP proteins in LNCaP cells (Figure 7A). Knockdown of YAP (Figure 7B) reduced the migration of LNCaP cells (Figure 7C). DHT increased the migration of LNCaP cells, while knockdown of YAP partially eliminated the androgenic induction of migration (Figure 7C). Treatment with YAP inhibitor verteporfin repressed the migration of PC‐3^AR^ and LNCaP cells in the absence of androgen as well as eliminated the androgenic induction of migration in both PC‐3^AR^ and LNCaP cells (Figure 7D). We examined the correlation between the gene expression level of AR and YAP1 in TCGA prostate cancer database (*n* = 498). We observed that AR gene level positively correlated with YAP1 gene level (*r* = 0.4959) (Figure 7E) in prostate tumors.

### 3.4 Androgen regulates EMT‐related miRNAs in PCa cells

**FIGURE 8:**
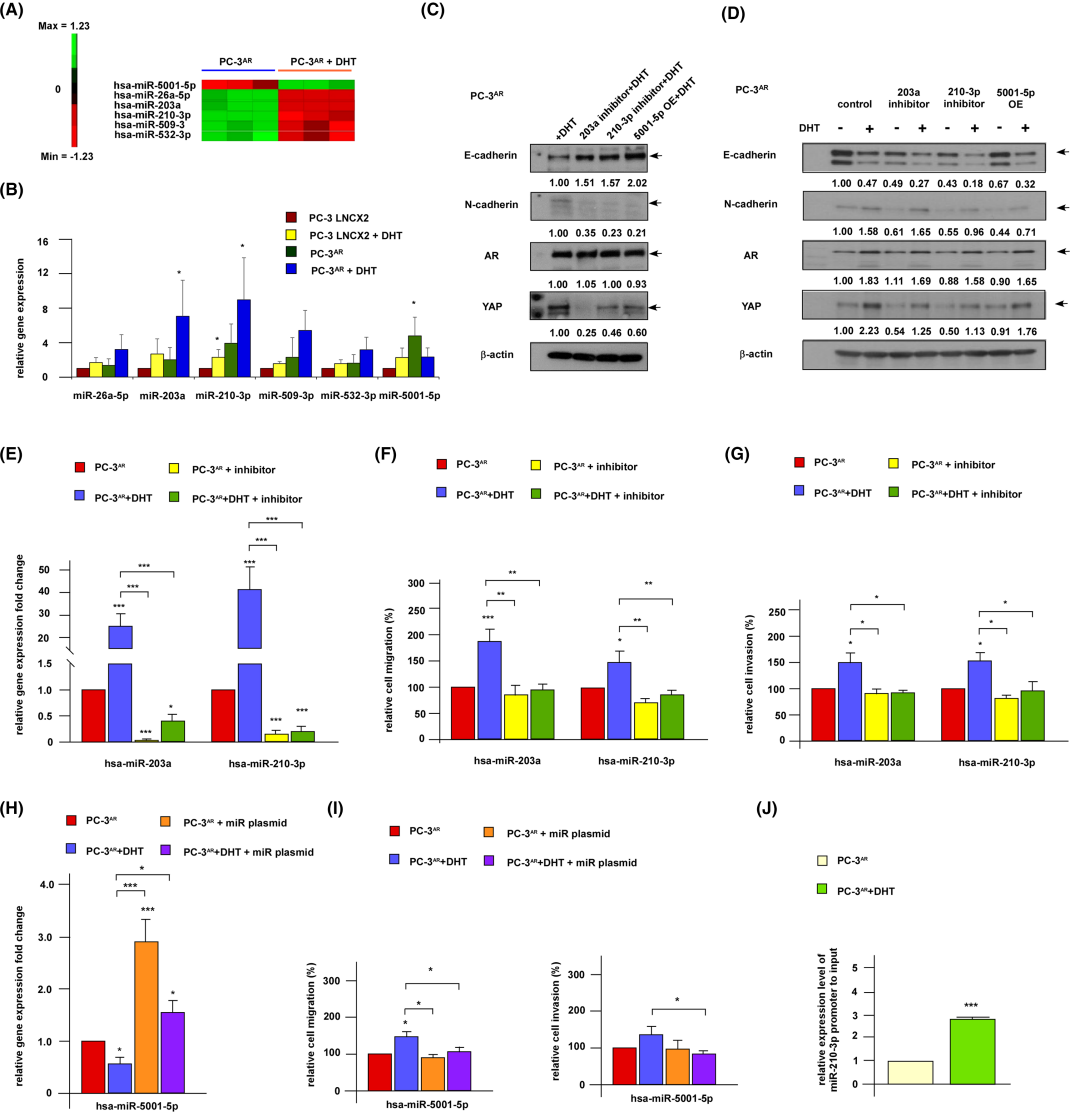
Expression of hsa‐miR‐5001‐5p, hsa‐miR‐203a and hsa‐miR‐210‐3p is regulated by androgen and alterations of these miRNAs regulate migration and invasion of PCa cells. (A) The miRNA array was used to examine the effect of androgen treatment on expression of miRNAs in PC‐3 and PC‐3^AR^ cells being treated with or without 10 nM dihydrotestosterone (DHT) for 48 h. The expression level of miRNAs was demonstrated using log_2_ value. (B) Effect of androgen treatment on expression of hsa‐miR‐5001‐5p, hsa‐miR‐26a‐5p, hsa‐miR‐203a, hsa‐miR‐210‐3p, hsa‐509‐3, and hsa‐miR‐532‐3p in PC‐3 and PC‐3^AR^ cells with or without 10 nM DHT for 48 h was examined by qRT‐PCR. (C) Protein expression of E‐cadherin, N‐cadherin, AR, and YAP in PC‐3^AR^ cells being treated with 10 nM DHT (C) or with or without 10 nM DHT (D) as well as inhibitor targeting hsa‐miR‐203a or hsa‐miR‐210‐3p, or overexpression plasmid for hsa‐miR‐5001‐5p for 48 h was assayed. The expression of hsa‐miR‐203a and hsa‐miR‐210‐3p (E), the migration (F), and invasion (G) in PC‐3^AR^ cells being treated with or without inhibitor was examined by qRT‐PCR and transwell assay, respectively. The expression of hsa‐miR‐5001‐5p (H), the migration and invasion (I) in PC‐3^AR^ cells being transfected with or without overexpression plasmid of hsa‐miR‐5001‐5p in the presence of absence of 10 nM DHT for 24 h was examined by qRT‐PCR and transwell assay, respectively (J) Binding of AR with promoter of miR210‐3p in PC‐3AR cells being treated with 0 or 10 nM DHT as determined by chromatin immunoprecipitation (ChIP) assay. *, **, *** represent statistically significant difference *p* < 0.05, *p* < 0.01, *p* < 0.001 respectively.

We suspected that miRNAs regulate the androgen/AR/YAP‐induced migration/invasion in PCa cells. We used miRNA array to determine if androgen and AR affects EMT proteins and YAP signaling proteins via regulation of miRNAs. Androgen decreased the expression of 38 hsa‐miRs but increased the expression of 12 hsa‐miRs (Figure S2). We are interested in the miRNAs which the expression levels were altered by androgen for at least 2 folds in PC‐3^AR^ cells but not in AR‐negative PC‐3 cells, because the expression of these miRNAs was regulated by androgen via AR. The hsa‐miR‐5001‐5p, hsa‐miR‐26a‐5p, hsa‐miR‐203a, hsa‐miR‐210‐3p, hsa‐miR‐509‐3, and hsa‐miR‐532‐3p fulfilled the requirement (Figure 8A). The qRT‐PCR showed that androgen decreased the expression of hsa‐miR‐5001‐5p but increased the expression of hsa‐miR‐203a and hsa‐miR‐210‐3p in PC‐3^AR^ cells (Figure 8B). The effect of androgen on other miRNAs was not statistically significant as determined by qRT‐PCR.

### 3.5 Alteration of miRNAs regulates EMT regulatory proteins and YAP in PCa cells

To examine if changes of hsa‐miR‐203a, hsa‐miR‐210‐3p, or hsa‐miR‐5001‐5p affects the expression of EMT regulatory proteins and YAP, we treated PC‐3^AR^ cells with hsa‐miR‐203a inhibitor, hsa‐miR‐210‐3p inhibitor, or transfection with overexpressing plasmid of hsa‐miR‐5001‐5p. Treatment with inhibitors targeting hsa‐miR‐203a or hsa‐miR‐210‐3p, or overexpression of hsa‐miR‐5001‐5p decreased the protein expression of androgen‐induced N‐cadherin and YAP but increased the abundance of androgen‐repressed E‐cadherin protein (Figure 8C,D). These effects were compromised by DHT (Figure 8D). Our observations suggested that androgen treatment induced YAP and N‐cadherin while suppressed E‐cadherin, at least partially, via regulation of hsa‐miR‐203a, hsa‐miR‐210‐3p, and hsa‐miR‐5001‐5p in AR‐positive PCa cells.

### 3.6 Androgen enhances PCa cancer metastasis via regulation of miRNAs

To explore if androgen promotes cell mobility and invasiveness of PCa cells via regulation of hsa‐miR‐203a, hsa‐miR‐210‐3p, or hsa‐miR‐5001‐5p, we examined the effects of inhibitor sequence targeting hsa‐miR‐203a or hsa‐miR‐210‐3p, as well as overexpression plasmid of hsa‐miR‐5001‐5p on cell movement and invasion of PCa cells. Androgen increased expression of hsa‐miR‐203a and hsa‐miR‐210‐3p, while treatment with inhibitors significantly suppressed the expression level of hsa‐miR‐203a and hsa‐miR‐210‐3p (Figure 8E). Inhibition of hsa‐miR‐203a or hsa‐miR‐210‐3p suppressed the androgen‐induced migration (Figure 8F) and invasion (Figure 8G) in PC‐3^AR^ cells. On the other hand, androgen decreased expression of hsa‐miR‐5001‐5p and overexpression plasmid increased the expression of hsa‐miR‐5001‐5p (Figure 8H). Elevation of hsa‐miR‐5001‐5p suppressed the androgen‐induced migration and invasion (Figure 8I) in PC‐3^AR^ cells.

As expression levels of these miRNA were affected by androgen, we suspected that there is androgen response element (ARE) within the promoter region of these miRNA gene sequence. Using PROMO computational website (http://alggen.lsi.upc.es/cgi‐bin/promo_v3/promo/promoinit.cgi?dirDB=TF_8.3), we identified three potential ARE sequences in the promoter region of hsa‐miR‐203a, four potential ARE sequences in the promoter region of miR‐210‐3p, and two potential ARE sequences in the promoter region of hsa‐miR‐5001 (Figure S3). These ARE elements may be involved in the androgenic regulation of hsa‐miR‐203a, hsa‐miR‐210‐3p and hsa‐miR‐5001. A ChIP assay demonstrated that AR binds with promoter region of has‐miR‐210‐3p in the presence of androgen, suggesting that androgen can regulate has‐miR‐210‐3p via AR binding to ARE of has‐miR‐210‐3p (Figure 8J).

## 4 DISCUSSION

In this study, we demonstrated that hsa‐miR‐203a, hsa‐miR‐210‐3p, and hsa‐miR‐5001 regulated the androgen/AR/YAP‐induced PCa metastasis. We discovered that the expression of hsa‐miR‐203a and hsa‐miR‐210‐3p was enhanced by androgen while androgen decreased the expression of hsa‐miR‐5001‐5p. All three of these miRNAs contained potential ARE region. ChIP assay demonstrated that AR binds with promoter region of has‐miR‐210‐3p. Inhibition of hsa‐miR‐203a or hsa‐miR‐210‐3p, or overexpression of hsa‐miR‐5001‐5p suppressed the migration and invasion of PCa cells. Our observation pointed out that expression of miR‐210‐3p was activated by androgen and promoted PCa metastasis, while knockdown of miR‐210‐3p decreased the expression of AR and YAP in PCa cells. The miR‐210‐3p has previously been reported to be increased in bone metastatic PCa and elevation of miR‐210‐3p was found positively correlated with high PSA levels, high Gleason grade, and bone metastasis in PCa patients.^20^ Overexpression of miR‐210‐3p was observed to activate NF‐κB signaling and to stimulate EMT, migration and invasion of PCa cells, while transfection of anti‐miR‐210‐3p increased survival and decreased PC‐3 bone metastasis in murine model of left cardiac ventricle inoculation.^20^ Secreted miR‐210‐3p reduced sensitivity of PCa cells to docetaxel treatment.^21^ We believe that miR‐210‐3p plays an important role in promoting PCa metastasis. The miR‐210‐3p has also been found to regulate metastasis of other cancers. For example, miR‐210‐3p increased the secreted exosomes from hypoxic neuroblastoma cells which stimulated the migration and invasion of neuroblastoma.^22^ The HIF‐1α/miR‐210‐3p/CPEB2 signaling axis regulated the EMT and metastasis of hepatocellular carcinoma.^23^ Lung cancer stem cell‐derived exosomal miR‐210‐3p bound fibroblast growth factor receptor‐like 1 and promoted metastasis of lung cancer.^24^ Hypoxia upregulated miR‐210‐3p and promoted EMT in glioma cells.^25^ For miR‐203, our results suggested a stimulating role of miR‐203 on PCa metastasis. However, some previous studies reported an anti‐metastatic role of miR‐203 in PCa cells. Overexpression of miR‐203 in PC‐3 cells was found to attenuate the development of bone metastasis in murine model.^26^ Activated AR was reported to activate miR‐203, which bound SRC and reduced stability of SRC, resulting in reduction of cell proliferation and migration in PCa cells.^27^ The opposite effects of miR‐203 in these two studies as compared to our current result may be due to the fact that AR‐negative PC‐3 was used in^26^ and the migration of LNCaP cells in^27^ was suppressed by androgen. Most other studies reported that androgen can enhance migration of LNCaP cells,^6^, ^28^, ^29^ which was consistent with our observation that androgen increased motility and invasiveness of PCa cells. We believed that miR‐203a acted as an inducer for PCa metastasis when androgen was present. Long non‐coding RNA (LncRNA) GAS5 was observed to repress the osteosarcoma metastasis via sponging miR‐203a, which regulated PI3K‐AKT signaling in osteosarcoma.^30^ SOX9 promoted esophageal cancer progression via activation of PI3K‐Akt pathway through miR‐203a.^31^ We suspected that the miR‐203a may enhance the migration and invasion of PCa cells via activation of PI3K‐Akt signaling. The role of miR‐5001‐5p in PCa is unclear. However, previous study showed an anticancer effect of miR‐5001‐5p in colorectal cancer. Long non‐coding RNA CCMAlnc downregulated miR‐5001‐5p and overexpression of CCMAlnc increased the proliferation and invasion of colorectal cancer cells.^32^ It will be interesting to trace the expression of these miRNAs during the disease progression of PCa.

We demonstrated that AR has dual and opposing role on migration and invasion of PCa cells, depending on the status of androgen and YAP. Residual androgens were found in the microenvironment of ADT‐treated prostate tissues.^33^, ^34^ Metastatic CRPC tumors exhibit higher intracrine steroidogenesis as well as higher levels of steroidogenic enzymes and AKR1C3.^35^, ^36^, ^37^ According to our current study, these residual androgen in tumor microenvironment can promote metastasis of AR‐positive PCa cells. Indeed, PCa patients expressing detectable PSA 12 months after the ending of ADT showed increased risk of metastasis.^38^ Additionally, enzalutamide treatment for PCa patients with high PSA level led to a 71% decrease of the risk of metastasis or death,^39^ which was consistent to our observation that AR suppressed PCa metastasis in androgen‐depleted condition.

In this study, we showed that YAP is essential for androgen/AR‐induced PCa metastasis. YAP is important in regulation of cell development^40^ and EMT.^41^, ^42^ Upon activation of the Hippo pathway, YAP is phosphorylated on serine 127, which prevents its translocation into the nucleus.^43^ WW/SH3 domain of YAP directly interacts with N‐terminal domain of AR^44^ and the AR‐YAP interaction is suppressed by MST1 via the regulation of Ser217 on YAP.^45^ Androgen enhances the interaction between AR and YAP, while antiandrogen enzalutamide attenuated the AR‐YAP interaction.^44^ YAP‐mediated G protein‐coupled estrogen receptor signaling is essential in regulation of proliferation and survival of prostate epithelium in benign prostatic hyperplasia.^46^ AR regulates YAP translation and AR‐mediated YAP activation is regulated by the RhoA GTPases transcriptional mediator, serum response factor (SRF).^47^ Our observations revealed that androgen suppressed the protein level of LATS1 and phosphorylation of YAP on Ser217, increased the nuclear accumulation of YAP, and enhanced the binding between AR and YAP. YAP transcriptionally activates Slug by binding to Transcriptional Enhanced Associate Domains (TEAD).^48^ Slug and AR reciprocally regulates each other.^49^ As we observed that AR and YAP accumulate in the nucleus when androgen is present, AR and YAP can transcriptionally activate the expression of SNAI1 and SNAI2 genes and therefore stimulate PCa metastasis. CDK5 has been found to affect the level of nuclear YAP and migration of melanoma cells.^50^ The induction of CDK5 by androgen observed in our study may thus increase the level of nuclear YAP and contributes to PCa metastasis. It was interesting that we observed knockdown of YAP in PC‐3^AR^ cells reduced the protein level of AR, this has not been reported before. However, we did not observe an increase of AR protein in PC‐3^AR^ cells overexpressing YAP. It is possible that YAP directly or indirectly regulates the phosphorylation of AR and thus regulates the stability of AR protein. Knockdown of YAP will reduce the stability of AR protein and thus decrease the AR protein level. We also believe that YAP does not regulate the transcription of AR, therefore overexpression of YAP will not increase the transcription of AR mRNA and will not increase the AR protein level.

## 5 CONCLUSIONS

In conclusion, we demonstrated that miRNAs 203a/210‐3p/5001‐5p regulate the androgen/AR/YAP‐induced PCa metastasis. These miRNAs and YAP can be useful targets for treating advanced PCa.

## AUTHOR CONTRIBUTIONS

**Chieh Huo:** Data curation (equal); formal analysis (equal); investigation (equal); methodology (equal); validation (equal); visualization (equal); writing – review and editing (equal). **Ying‐Yu Kuo:** Data curation (equal); formal analysis (equal); investigation (equal); validation (equal); visualization (equal). **Ching‐Yu Lin:** Data curation (equal); investigation (equal); visualization (equal); writing – review and editing (equal). **Shine‐Gwo Shiah:** Conceptualization (equal); investigation (equal); methodology (equal); resources (equal); writing – review and editing (equal). **Chia‐Yang Li:** Formal analysis (equal); validation (equal); visualization (equal); writing – review and editing (equal). **Shu‐Pin Huang:** Formal analysis (equal); validation (equal); visualization (equal); writing – review and editing (equal). **Jen‐Kun Chen:** Formal analysis (equal); validation (equal); visualization (equal); writing – review and editing (equal). **Wen‐Ching Wang:** Conceptualization (equal); methodology (equal); validation (equal); writing – review and editing (equal). **Hsing‐Jien Kung:** Conceptualization (equal); methodology (equal); resources (equal); validation (equal); writing – review and editing (equal). **Chih‐Pin Chuu:** Conceptualization (equal); formal analysis (equal); funding acquisition (equal); methodology (equal); project administration (equal); resources (equal); supervision (equal); validation (equal); visualization (equal); writing – original draft (equal); writing – review and editing (equal).

## FUNDING INFORMATION

This study was supported by MOST 105‐2628‐B‐400‐005‐MY3, MOST 109‐2320‐B‐400‐004‐MY3, NSTC 112‐2314‐B‐400‐031, NSTC 112‐2320‐B‐400‐008 from Ministry of Science and Technology (MOST) and NHRIKMU‐111‐I002, CS‐109‐PP‐03, CS‐112‐PP‐03, CS‐113‐PP‐03 from National Health Research Institutes (NHRI) for CPC; NSTC 112‐2326‐B‐038‐001 from National Science and Technology Council (NSTC) for HJK. CYL was supported by NSTC 112‐2326‐B‐038‐001, while CH was supported by NSTC 113‐2811‐B‐400‐024 (NSTC).

## CONFLICT OF INTEREST STATEMENT

The authors declare no conflict of interest.

## ETHICS STATEMENT

Approval of the research protocol by an Institutional Reviewer Board: N/A. Animal Studies: N/A.

## Supporting information


**Figure S1.** Effects of castration on angiogenesis and metastasis of PC‐3 and PC‐3^AR^ xenograft in nude mice. PC‐3 LNCX2 control cells and PC‐3^AR^ cells were injected at 10^6^ cells/side into both flanks of castrated nude mice or intact nude mice. Tumor cells were allowed to grow for 40 days and mice were sacrificed. Angiogenesis and metastasis of PC‐3 LNCX2 tumors and PC‐3^AR^ tumors were examined at the time of sacrifice. The animal protocol 111,111‐AC1‐M1 was approved by NHRI IACUC.

**Figure S2.** Heatmap demonstrates the alteration of miRNAs expression in PC‐3AR cells with or without 10 nM DHT. The mean value of the expression of three repeats of the specific miRNAs in PC‐3^AR^ cells without DHT was set to be 0. Each repeat of the specific miRNA in PC‐3^AR^ cells with DHT was then expressed as the relative value shown in log_2_.

**Figure S3.** Potential ARE elements in the promoter regions of miRNAs. We used PROMO computational website (http://alggen.lsi.upc.es/cgi‐bin/promo_v3/promo/promoinit.cgi?dirDB=TF_8.3) and identified three potential ARE sequence in the promoter region of hsa‐miR‐203a, four potential ARE sequences in the promoter region of miR‐210‐3p, and two potential ARE sequences in the promoter region of hss‐miR‐5001. The sequences and locations of these ARE were shown.

**Table S1.** Information of antibodies used in micro‐western array (MWA) analysis. Information of each antibody being used in MWA analysis was listed.

**Data S1.** Supporting Information.

## Data Availability

The data that supports the findings of this study are available in the supplementary material of this article.

## References

[1] Levesque MH , El‐Alfy M , Cusan L , Labrie F . Androgen receptor as a potential sign of prostate cancer metastasis. Prostate. 2009;69:1704‐1711. 19670238 10.1002/pros.21021

[2] Chen Y , Lin Y , Nie P , et al. Associations of prostate‐specific antigen, prostate carcinoma tissue Gleason score, and androgen receptor expression with bone metastasis in patients with prostate carcinoma. Med Sci Monit. 2017;23:1768‐1774. 28400549 10.12659/MSM.900977 PMC5398423

[3] Lin CY , Jan YJ , Kuo LK , et al. Elevation of androgen receptor promotes prostate cancer metastasis by induction of epithelial‐mesenchymal transition and reduction of KAT5. Cancer Sci. 2018;109:3564‐3574. 30142696 10.1111/cas.13776 PMC6215884

[4] Zheng Y , Li P , Huang H , et al. Androgen receptor regulates eIF5A2 expression and promotes prostate cancer metastasis via EMT. Cell Death Dis. 2021;7:373. 10.1038/s41420-021-00764-x PMC8643356 34864817

[5] Lucas JM , Heinlein C , Kim T , et al. The androgen‐regulated protease TMPRSS2 activates a proteolytic cascade involving components of the tumor microenvironment and promotes prostate cancer metastasis. Cancer Discov. 2014;4:1310‐1325. 25122198 10.1158/2159-8290.CD-13-1010 PMC4409786

[6] Ko CJ , Huang CC , Lin HY , et al. Androgen‐induced TMPRSS2 activates matriptase and promotes extracellular matrix degradation, prostate cancer cell invasion, tumor growth, and metastasis. Cancer Res. 2015;75:2949‐2960. 26018085 10.1158/0008-5472.CAN-14-3297

[7] Tousignant KD , Rockstroh A , Taherian Fard A , et al. Lipid uptake is an androgen‐enhanced lipid supply pathway associated with prostate cancer disease progression and bone metastasis. Mol Cancer Res. 2019;17:1166‐1179. 30808729 10.1158/1541-7786.MCR-18-1147

[8] Huo C , Kao YH , Chuu CP . Androgen receptor inhibits epithelial‐mesenchymal transition, migration, and invasion of PC‐3 prostate cancer cells. Cancer Lett. 2015;369:103‐111. 26297988 10.1016/j.canlet.2015.08.001

[9] Xie H , Li L , Zhu G , et al. Infiltrated pre‐adipocytes increase prostate cancer metastasis via modulation of the miR‐301a/androgen receptor (AR)/TGF‐beta1/Smad/MMP9 signals. Oncotarget. 2015;6:12326‐12339. 25940439 10.18632/oncotarget.3619 PMC4494941

[10] Jennbacken K , Tesan T , Wang W , Gustavsson H , Damber JE , Welen K . N‐cadherin increases after androgen deprivation and is associated with metastasis in prostate cancer. Endocr Relat Cancer. 2010;17:469‐479. 20233707 10.1677/ERC-10-0015

[11] Chen WY , Tsai YC , Yeh HL , et al. Loss of SPDEF and gain of TGFBI activity after androgen deprivation therapy promote EMT and bone metastasis of prostate cancer. Sci Signal. 2017;10:eaam6826. 28811384 10.1126/scisignal.aam6826

[12] Oh‐Hohenhorst SJ , Lange T . Role of metastasis‐related microRNAs in prostate cancer progression and treatment. Cancers (Basel). 2021;13:4492. 34503302 10.3390/cancers13174492 PMC8431208

[13] Takayama KI , Misawa A , Inoue S . Significance of microRNAs in androgen signaling and prostate cancer progression. Cancers (Basel). 2017;9:102. 28783103 10.3390/cancers9080102 PMC5575605

[14] Chuu CP , Chen RY , Hiipakka RA , et al. The liver X receptor agonist T0901317 acts as androgen receptor antagonist in human prostate cancer cells. Biochem Biophys Res Commun. 2007;357:341‐346. 17416342 10.1016/j.bbrc.2007.03.116 PMC2693411

[15] Horwitz KB , Koseki Y , McGuire WL . Estrogen control of progesterone receptor in human breast cancer: role of estradiol and antiestrogen. Endocrinology. 1978;103:1742‐1751. 748014 10.1210/endo-103-5-1742

[16] Chuu CP , Lin HP , Ciaccio MF , et al. Caffeic acid phenethyl ester suppresses the proliferation of human prostate cancer cells through inhibition of p70S6K and Akt signaling networks. Cancer Prev Res (Phila). 2012;5:788‐797. 22562408 10.1158/1940-6207.CAPR-12-0004-T PMC4962698

[17] Tseng JC , Huang SH , Lin CY , et al. ROR2 suppresses metastasis of prostate cancer via regulation of miR‐199a‐5p‐PIAS3‐AKT2 signaling axis. Cell Death Dis. 2020;11:376. 32415173 10.1038/s41419-020-2587-9 PMC7228945

[18] Ciaccio MF , Wagner JP , Chuu CP , Lauffenburger DA , Jones RB . Systems analysis of EGF receptor signaling dynamics with microwestern arrays. Nat Methods. 2010;7:148‐155. 20101245 10.1038/nmeth.1418 PMC2881471

[19] Zhao B , Li L , Tumaneng K , Wang CY , Guan KL . A coordinated phosphorylation by Lats and CK1 regulates YAP stability through SCF(beta‐TRCP). Genes Dev. 2010;24:72‐85. 20048001 10.1101/gad.1843810 PMC2802193

[20] Ren D , Yang Q , Dai Y , et al. Oncogenic miR‐210‐3p promotes prostate cancer cell EMT and bone metastasis via NF‐kappaB signaling pathway. Mol Cancer. 2017;16:117. 28693582 10.1186/s12943-017-0688-6 PMC5504657

[21] Canovai M , Evangelista M , Mercatanti A , et al. Secreted miR‐210‐3p, miR‐183‐5p and miR‐96‐5p reduce sensitivity to docetaxel in prostate cancer cells. Cell Death Dis. 2023;9:445. 10.1038/s41420-023-01696-4 PMC10709610 38065937

[22] Fusco P , Fietta A , Esposito MR , et al. miR‐210‐3p enriched extracellular vesicles from hypoxic neuroblastoma cells stimulate migration and invasion of target cells. Cell Biosci. 2023;13:89. 37202777 10.1186/s13578-023-01045-z PMC10193740

[23] You R , Yang Y , Yin G , et al. CPEB2 suppresses hepatocellular carcinoma epithelial‐mesenchymal transition and metastasis through regulating the HIF‐1alpha/miR‐210‐3p/CPEB2 Axis. Pharmaceutics. 2023;15:1887. 37514073 10.3390/pharmaceutics15071887 PMC10386397

[24] Wang L , He J , Hu H , et al. Lung CSC‐derived exosomal miR‐210‐3p contributes to a pro‐metastatic phenotype in lung cancer by targeting FGFRL1. J Cell Mol Med. 2020;24:6324‐6339. 32396269 10.1111/jcmm.15274 PMC7294132

[25] Liu H , Chen C , Zeng J , Zhao Z , Hu Q . MicroRNA‐210‐3p is transcriptionally upregulated by hypoxia induction and thus promoting EMT and chemoresistance in glioma cells. PLoS One. 2021;16:e0253522. 34197482 10.1371/journal.pone.0253522 PMC8248614

[26] Saini S , Majid S , Yamamura S , et al. Regulatory role of mir‐203 in prostate cancer progression and metastasis. Clin Cancer Res. 2011;17:5287‐5298. 21159887 10.1158/1078-0432.CCR-10-2619

[27] Siu MK , Chen WY , Tsai HY , et al. Androgen receptor regulates SRC expression through microRNA‐203. Oncotarget. 2016;7:25726‐25741. 27028864 10.18632/oncotarget.8366 PMC5041939

[28] Chuan YC , Pang ST , Cedazo‐Minguez A , Norstedt G , Pousette A , Flores‐Morales A . Androgen induction of prostate cancer cell invasion is mediated by ezrin. J Biol Chem. 2006;281:29938‐29948. 16873375 10.1074/jbc.M602237200

[29] Frigo DE , Sherk AB , Wittmann BM , et al. Induction of Kruppel‐like factor 5 expression by androgens results in increased CXCR4‐dependent migration of prostate cancer cells in vitro. Mol Endocrinol. 2009;23:1385‐1396. 19460858 10.1210/me.2009-0010 PMC2737557

[30] Wang Y , Kong D . LncRNA GAS5 represses osteosarcoma cells growth and metastasis via sponging MiR‐203a. Cell Physiol Biochem. 2018;45:844‐855. 29414815 10.1159/000487178

[31] Wang L , Zhang Z , Yu X , et al. SOX9/miR‐203a axis drives PI3K/AKT signaling to promote esophageal cancer progression. Cancer Lett. 2020;468:14‐26. 31600529 10.1016/j.canlet.2019.10.004

[32] Yan Y , Xuan B , Gao Z , et al. CCMAlnc promotes the malignance of colorectal cancer by modulating the interaction between miR‐5001‐5p and its target mRNA. Front Cell Dev Biol. 2020;8:566932. 33681178 10.3389/fcell.2020.566932 PMC7931267

[33] van der Sluis TM , Vis AN , van Moorselaar RJ , et al. Intraprostatic testosterone and dihydrotestosterone. Part I: concentrations and methods of determination in men with benign prostatic hyperplasia and prostate cancer. BJU Int. 2012;109:176‐182. 21992222 10.1111/j.1464-410X.2011.10651.x

[34] Mostaghel EA , Page ST , Lin DW , et al. Intraprostatic androgens and androgen‐regulated gene expression persist after testosterone suppression: therapeutic implications for castration‐resistant prostate cancer. Cancer Res. 2007;67:5033‐5041. 17510436 10.1158/0008-5472.CAN-06-3332

[35] Montgomery RB , Mostaghel EA , Vessella R , et al. Maintenance of intratumoral androgens in metastatic prostate cancer: a mechanism for castration‐resistant tumor growth. Cancer Res. 2008;68:4447‐4454. 18519708 10.1158/0008-5472.CAN-08-0249 PMC2536685

[36] Stanbrough M , Bubley GJ , Ross K , et al. Increased expression of genes converting adrenal androgens to testosterone in androgen‐independent prostate cancer. Cancer Res. 2006;66:2815‐2825. 16510604 10.1158/0008-5472.CAN-05-4000

[37] Mitsiades N , Sung CC , Schultz N , et al. Distinct patterns of dysregulated expression of enzymes involved in androgen synthesis and metabolism in metastatic prostate cancer tumors. Cancer Res. 2012;72:6142‐6152. 22971343 10.1158/0008-5472.CAN-12-1335 PMC3685485

[38] Lim DM , Gulati R , Aleshin‐Guendel S , et al. Undetectable prostate‐specific antigen after short‐course androgen deprivation therapy for biochemically recurrent patients correlates with metastasis‐free survival and prostate cancer‐specific survival. Prostate. 2018;78:1077‐1083. 10.1002/pros.23666 PMC6328347 29987912

[39] Hussain M , Fizazi K , Saad F , et al. Enzalutamide in men with nonmetastatic, castration‐resistant prostate cancer. N Engl J Med. 2018;378:2465‐2474. 29949494 10.1056/NEJMoa1800536 PMC8288034

[40] Wang K , Degerny C , Xu M , Yang XJ . YAP, TAZ, and Yorkie: a conserved family of signal‐responsive transcriptional coregulators in animal development and human disease. Biochem Cell Biol. 2009;87:77‐91. 19234525 10.1139/O08-114

[41] Shao DD , Xue W , Krall EB , et al. KRAS and YAP1 converge to regulate EMT and tumor survival. Cell. 2014;158:171‐184. 24954536 10.1016/j.cell.2014.06.004 PMC4110062

[42] Zhang J , Ji JY , Yu M , et al. YAP‐dependent induction of amphiregulin identifies a non‐cell‐autonomous component of the Hippo pathway. Nat Cell Biol. 2009;11:1444‐1450. 19935651 10.1038/ncb1993 PMC2819909

[43] Johnson R , Halder G . The two faces of hippo: targeting the Hippo pathway for regenerative medicine and cancer treatment. Nat Rev Drug Discov. 2014;13:63‐79. 24336504 10.1038/nrd4161 PMC4167640

[44] Kuser‐Abali G , Alptekin A , Lewis M , Garraway IP , Cinar B . YAP1 and AR interactions contribute to the switch from androgen‐dependent to castration‐resistant growth in prostate cancer. Nat Commun. 2015;6:8126. 28230103 10.1038/ncomms9126 PMC5327734

[45] Ni L , Llewellyn R , Kesler CT , et al. Androgen induces a switch from cytoplasmic retention to nuclear import of the androgen receptor. Mol Cell Biol. 2013;33:4766‐4778. 24100013 10.1128/MCB.00647-13 PMC3889559

[46] Liu Z , Li S , Chen S , et al. YAP‐mediated GPER signaling impedes proliferation and survival of prostate epithelium in benign prostatic hyperplasia. iScience. 2024;27:109125. 38420594 10.1016/j.isci.2024.109125 PMC10901089

[47] Salem O , Jia S , Qian BZ , Hansen CG . AR activates YAP/TAZ differentially in prostate cancer. Life Sci Alliance. 2023;6:e202201620. 37385752 10.26508/lsa.202201620 PMC10310930

[48] Yu M , Chen Y , Li X , et al. YAP1 contributes to NSCLC invasion and migration by promoting slug transcription via the transcription co‐factor TEAD. Cell Death Dis. 2018;9:464. 29700328 10.1038/s41419-018-0515-z PMC5920099

[49] Wu K , Gore C , Yang L , et al. Slug, a unique androgen‐regulated transcription factor, coordinates androgen receptor to facilitate castration resistance in prostate cancer. Mol Endocrinol. 2012;26:1496‐1507. 22745193 10.1210/me.2011-1360 PMC5416972

[50] Caron JM , Han X , Contois L , Vary CPH , Brooks PC . The HU177 collagen epitope controls melanoma cell migration and experimental metastasis by a CDK5/YAP‐dependent mechanism. Am J Pathol. 2018;188:2356‐2368. 30118657 10.1016/j.ajpath.2018.06.017 PMC6180252

