# Supplementary figures and images for: The miRNAs 203a/210‐3p/5001‐5p regulate the androgen/androgen receptor/YAP‐induced migration in prostate cancer cells
Source: Cancer Med. 2024 Aug 16;13(16):e70106. doi: 10.1002/cam4.70106 (PMC11327718; doi:10.1002/cam4.70106)

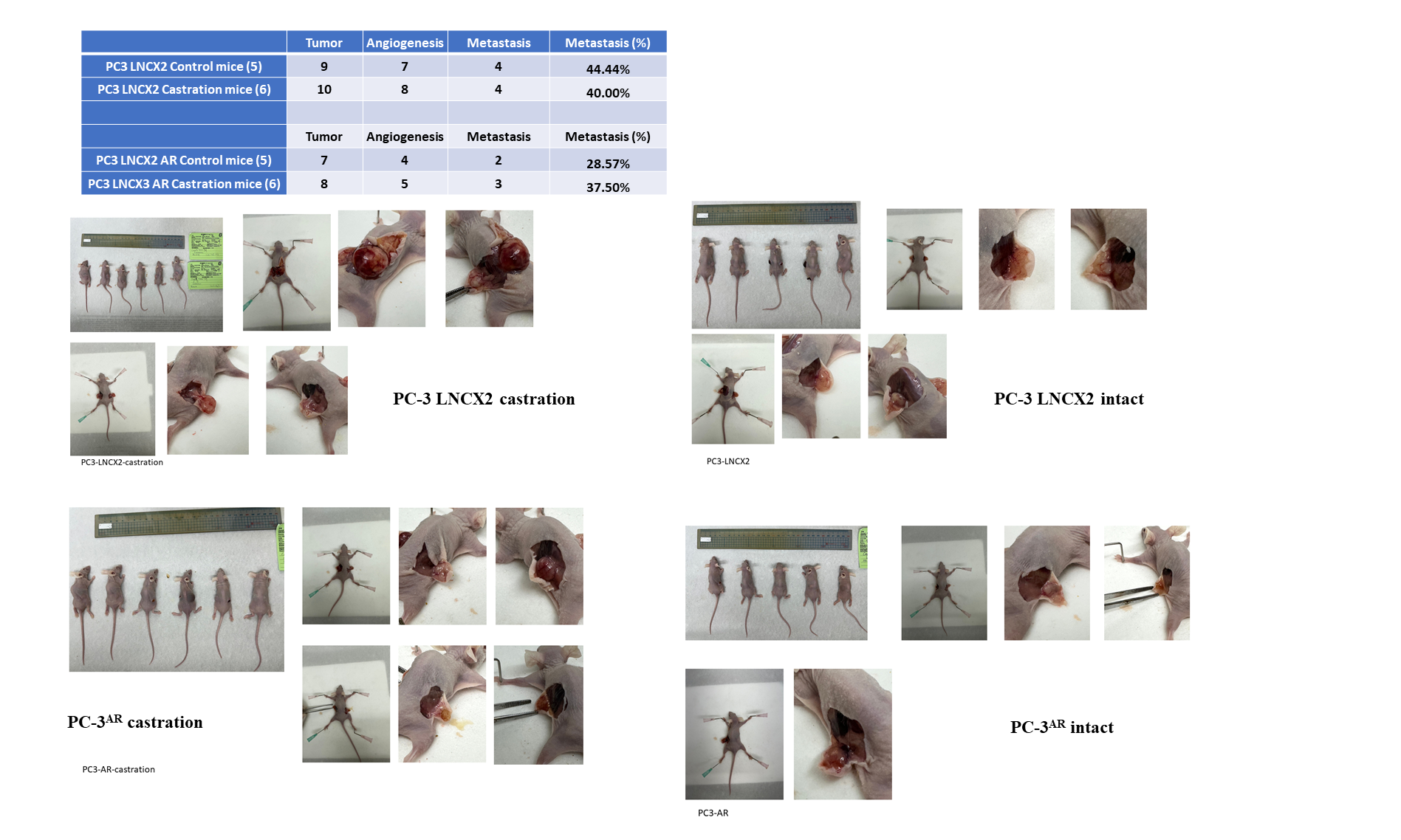

Supplement: Supplementary file 1 — Figure S1. Effects of castration on angiogenesis and metastasis of PC‐3 and PC‐3AR xenograft in nude mice. PC‐3 LNCX2 control cells and PC‐3AR cells were injected at 106 cells/side into both flanks of castrated nude mice or intact nude mice. Tumor cells were allowed to grow for 40 days and mice were sacrificed. Angiogenesis and metastasis of PC‐3 LNCX2 tumors and PC‐3AR tumors were examined at the time of sacrifice. The animal protocol 111,111‐AC1‐M1 was approved by NHRI IACUC. [file CAM4-13-e70106-s003.tif]

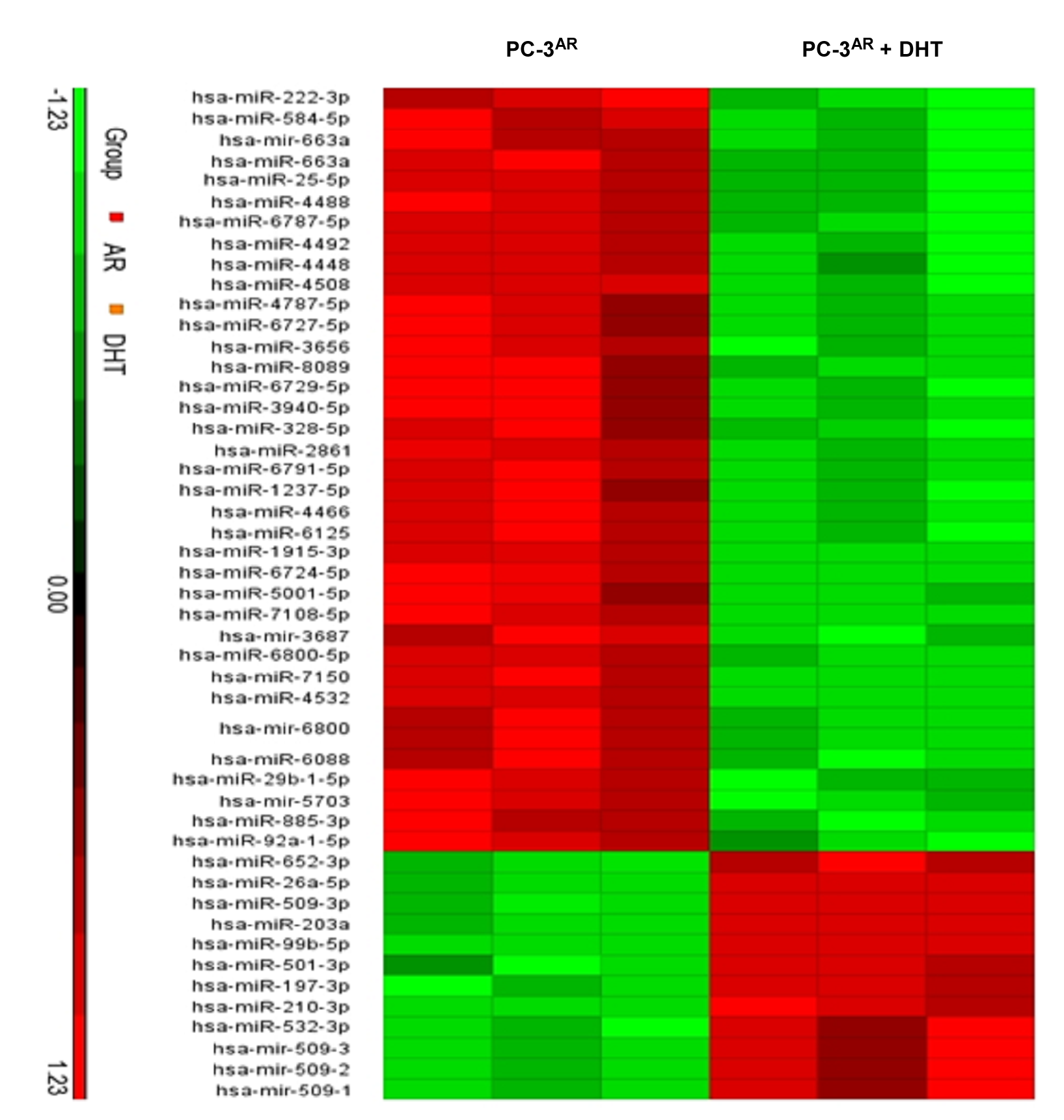

Supplement: Supplementary file 2 — Figure S2. Heatmap demonstrates the alteration of miRNAs expression in PC‐3AR cells with or without 10 nM DHT. The mean value of the expression of three repeats of the specific miRNAs in PC‐3AR cells without DHT was set to be 0. Each repeat of the specific miRNA in PC‐3AR cells with DHT was then expressed as the relative value shown in log2. [file CAM4-13-e70106-s005.tif]

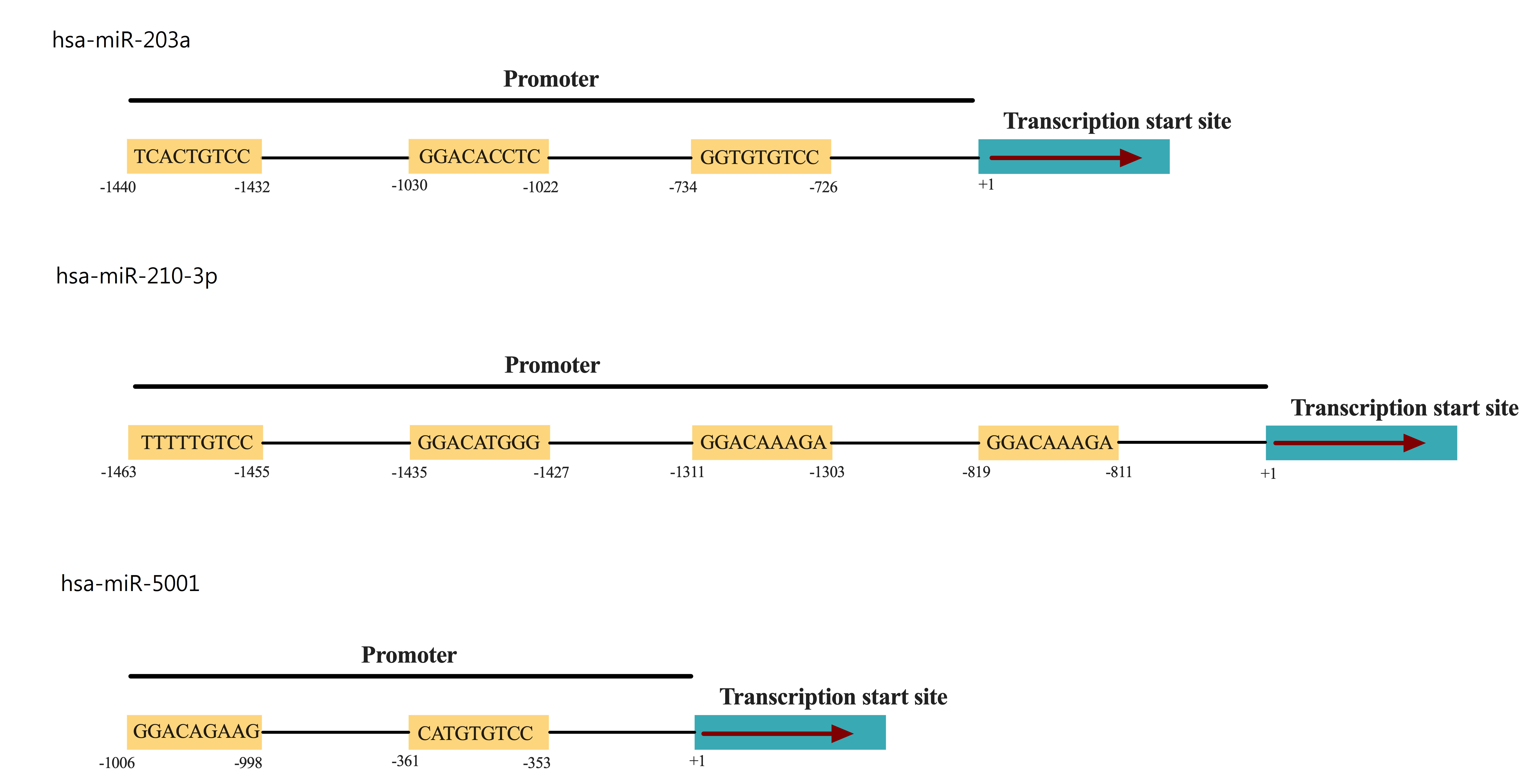

Supplement: Supplementary file 3 — Figure S3. Potential ARE elements in the promoter regions of miRNAs. We used PROMO computational website (http://alggen.lsi.upc.es/cgi‐bin/promo_v3/promo/promoinit.cgi?dirDB=TF_8.3) and identified three potential ARE sequence in the promoter region of hsa‐miR‐203a, four potential ARE sequences in the promoter region of miR‐210‐3p, and two potential ARE sequences in the promoter region of hss‐miR‐5001. The sequences and locations of these ARE were shown. [file CAM4-13-e70106-s004.tif]
