# Supplementary material for: The miRNAs 203a/210‐3p/5001‐5p regulate the androgen/androgen receptor/YAP‐induced migration in prostate cancer cells
Source: Cancer Med. 2024 Aug 16;13(16):e70106. doi: 10.1002/cam4.70106 (PMC11327718; doi:10.1002/cam4.70106)
Supplement: Supplementary file 4 — Table S1. Information of antibodies used in micro‐western array (MWA) analysis. Information of each antibody being used in MWA analysis was listed. [file CAM4-13-e70106-s001.docx]

| Antibody | Brand | Catalog |
| --- | --- | --- |
| ABCA1 | abcam | ab18180 |
| ABCG1 | abcam | ab52617 |
| APC | abcam | ab40778 |
| AR | abcam | ab108341 |
| β-actin | Novus | NB600-501 |
| β-catenin | Cell signaling | 9582S |
| c-Jun | Cell signaling | 9165S |
| c-Myc | abcam | ab32072 |
| CYP27A1 | abcam | ab126785 |
| E-cadherin | BD | 610182 |
| Frizzled | Cell signaling | 5266S |
| GAPDH | Novus | NB300-221 |
| GSK3𝛼 | Cell signaling | 4337S |
| GSK3β | Cell signaling | 9315S |
| Lamin A/C | GeneTex | GTX111677 |
| LXR𝛼 | Santa Cruz | sc-34386 |
| LXRβ | Santa Cruz | sc-133221 |
| MMP2 | abcam | ab37150 |
| MMP7 | Millipore | MAB3322 |
| MMP8 | abcam | ab53017 |
| MMP9 | abcam | ab76003 |
| N-cadherin | BD | 610921 |
| NF-_𝑘_B p50 | Millipore | 06-886 |
| NF-_𝑘_B p65 | Santa Cruz | sc-372 |
| p-AR | Santa Cruz | sc-71773 |
| phospho-β-catenin | Cell signaling |  |
| phospho-GSK3𝛼 (Ser21) | Upstate | 07-393 |
| phospho-GSK3β (Ser9) | Cell signaling | 9322S |
| PSA | Dako | A0562 |
| Ras | Millipore | 05-516 |
| slug | Cell signaling | 9585S |
| slug | Novus | NBP2-27182SS |
| Snail | abcam | ab53519 |
| snail | Cell signaling | 3879S |
| SREBF1 | Epitomics | T3402 |
| SREBP1 | abcam | ab3259 |
| TAZ | Cell signaling | 4883S |
| TMPRSS2 | Cell signaling | ab92323 |
| Twist1 | GeneTex | GTX127310 |
| VEGF Receptor 1 | abcam | ab32152 |
| VEGF Receptor 2 | abcam | ab134191 |
| VEGF Receptor 3 | abcam | ab27278 |
| VEGFA | abcam | ab183100 |
| VEGF-C | Cell signaling | 2445S |
| vimentin | Cell signaling | 5741S |
| Wnt-1 | Santa Cruz | sc-5630 |
| Wnt-3a | Santa Cruz | sc-136163 |
| Wnt5 a/b | Cell signaling | 2530S |
| YAP | Cell signaling | 4912S |
| Phospho-YAP S127 | Genetex | GTX130424 |
