# Supplementary material for: The miRNAs 203a/210‐3p/5001‐5p regulate the androgen/androgen receptor/YAP‐induced migration in prostate cancer cells
Source: Cancer Med. 2024 Aug 16;13(16):e70106. doi: 10.1002/cam4.70106 (PMC11327718; doi:10.1002/cam4.70106)
Supplement: Supplementary file 5 — Data S1. Supporting Information. [file CAM4-13-e70106-s002.docx]

**Supplemental Materials and Methods**

**Cell culture, Chemicals and Plasmids.** PC-3 and LNCaP FGC cells were from Bioresource Collection and Research Center (Hsinchu City, Taiwan). PC-3^AR^ cells were generated by transfecting PC-3 cells with LNCX-2 plasmid containing wild-type human AR and selected with neomycin G418 as previously described ^15^. All PCa cells were cultured in DMEM (Gibco/Invitrogen) supplemented with 10% charcoal-stripped fetal bovine serum (CS-FBS) ^16^. FBS was from Biological Industries, Beit Haemek, Israel. DHT was from Sigma Aldrich (St. Louis, MO, U.S.A).

**Zebrafish xenotransplantation assay.** For xenotransplantation assay, 9x10^5^ of trypsinized cells were resuspended in 1 ml PBS and labeled with 25mM carboxyfluorescein succinimidyl ester (CellTrace™ CFSE, Thermo Fisher). After CFSE incubation, the cells were washed by PBS twice and re-suspended in 20 μL PBS. The injection volume was 4.6 nL containing 300-400 PC-3^AR^ cells into the yolk sac of embryos at 2-dpf via glass capillary using a Nanoject II™ Nanoliter injector (Drummond Scientific, Broomall, PA, USA). Embryos were then incubated under gradually increasing of temperature from 28℃ to 37℃ either with 0.1% EtOH or 10 nM DHT stay at 37 ℃ for 1 day. To determine the metastatic distance, embryos bearing tumor cells were separated into a 96 Well Polystyrol Zellkultur Microplatte (Greiner Bio-One, Kremsmünster, Austria) and photos was taken by fluorescent microscope (ZEISS Discovery V8, 20X magnification). The fluorescent area of CFSE signal were quantified using MetaXpress (Molecular Devices). The transverse length of fluorescent signal was measured using ImageJ and the metastasis was clustered into Class 1 (cells with fluorescence dye appear in head, body, and tail blood vessels), Class 2 (cells with fluorescence dye appear in head and tail blood vessels as well as a small portion in the body), Class 3 (Only in the head and tail blood vessels) and Class 4 (the amount of fluorescent cells is very little amount or almost undetectable).

**The micro-RNA array.** We used 1000 ng of each sample to join a tail of Poly (A) with a PolyA Polymerase and labeled the samples with biotin following the protocol of FlashTag Biotin HSR RNA Labeling Kit for Applied Biosystems GeneChip miRNA arrays (Genisphere®). 21.5 μLof Biotin-labeled sample were hybridized for 16 h at 48℃ on GeneChip™ miRNA 4.0 Array. GeneChips were washed and stained in the Affymetrix Fluidics Station 450. GeneChips were scanned using the Affymetrix GeneArray Scanner 3000 7G. HeatMAP was drawed by Partek Genomics Suite.

**The miRNA extraction and qPCR analysis.** Trizol reagent (Themo fisher) was used for miRNA purification following manufacturer's protocol. 2 μg of total RNA was used as templates for miRNA cDNA synthesis by cDNA Synthesis kit (Origene, HP100042). The qPCR analysis was performed by Maxima SYBR Green/ROX qPCR Master Mix (2X) (Fermentas/Thermo Fisher). The miRNA expression was analyzed by ViiA 7 real-time PCR system (Applied Biosystems/Life Technologies, Waltham, Massachusetts, U.S.A.). The sequences used for qPCR primers were listed as following:

hsa-miR-26a-5p TTC AAG TAA TCC AGG ATA GGC

hsa-miR-203a GTG AAA TGT TTA GGA CCA CTA G

hsa-miR-210-3p CTG TGC GTG TGA CAG CGG CTG A

hsa-miR-509-3p TGA TTG GTA CGT CTG TGG GTA G

hsa-miR-532-3p CCT CCC ACA CCC AAG GCT TGC A

hsa-miR-5001-5p AGG GCT GGA CTC AGC GGC GGA GCT

**Real-Time Quantitative PCR.** PC-3 and PC-3^AR^ cells treated with or without 10 nM DHT were extracted for RNA with the RNeasy Mini Kit from Qiagen (Germantown, MD, USA) following the manufacturer’s instructions. The primer sequences were designed by Primer3, and the sequences were as follows: YAP forward: 5′-GGTGCCACTGTTAAGGAAAGG-3′ and reverse: 5′-GTGAGGCCACAGGAGTTAGC-3′; CTGF forward: 5′-TGGTGCAGCCAGAAAGCTC-3′ and reverse: 5′-CCAATGACAACGCCTCCTG-3′; Cyr61 forward: 5′-TTCTTTCACAAGGCGGCACTC-3′ and reverse: 5′-AGCCTCGCATCCTATACAACC-3′. Expression of GAPDH gene was used as loading control. Real-time quantitative PCR was performed by Maxima 2X SYBR Green/ROX, and 5 endogenous reference RNAs were as normalization signals. Raw data of CT value was analyzed by the ΔΔCT software in SBI website.

**Immunoblot analysis.** Information of antibodies detecting the specific proteins are listed as following: androgen receptor (AR) and c-Myc were from Abcam (Cambridge, MA, U.S.A); CDK1, CDK5, CDK9, β-catenin, c-Jun, GSK-3𝛼, Snail, Slug, TAZ, vimentin, and YAP were from Cell signaling (Danvers, MA, U.S.A); GAPDH was from Novus (Littleton, CO, U.S.A); E-cadherin and N-cadherin were from BD (Franklin Lakes, NJ, U.S.A); Twist1 was from Genetex (Irvine, CA, U.S.A); phospho-AR S81 and IgG were from Santa Cruz (Dallas, TX, U.S.A). The intensity of indicated Western blot bands were quantified by ImageJ software.

**Immunofluorescence.** Cells were seeded in millicell EZ slide (Millipore) and treated with DHT or not for 48 h. Cells were fixed with 4% para-formaldehyde on ice for 15 minutes and permeabilized in 0.3% Triton X-100 (in PBS) for 10 minutes. Samples were then blocked for an hour and were stained with indicated antibody for 16 h at 4 °C. Alexa Fluor 488 dye and Alexa Fluor 594 dye (Thermo Fisher Scientific) were used as secondary antibody for green and red fluorescent dye. The cell nuclei were stained with DAPI.

**Wound healing assay.** Cells which pre-treated with 10 nM DHT or control vehicle for 48 h and cells were then seeded at 4 × 10^4^ cells/100 μl into ibidi culture inserts in a 24-well plate. After 24 h, wound healing assay was performed with ibidi culture insert (Applied Biophysics, Troy, NY, U.S.A.) according to the manufacturer’s instructions. Cells were monitored and photographed with a live imaging microscope (Leica AF 6000 LX, Leica, Wetzlar, Germany).

**Co-immunoprecipitation.** Cells were lysed by Pierce IP Lysis buffer (Thermo Fisher Scientific) containing protease inhibitor, phosphatase inhibitor, and Na_2_VO_3_. Lysate was centrifuged for 10 minutes at 13000 rpm/4 °C and the pellet was discarded. Lysate was incubated with Protein G Sepharose beads and was washed by PBS twice. Appropriate amount of primary antibody/IgG and beads were mixed on shaker for 1 h at 4 °C. Beads were then washed by PBS twice. Total protein (1 mg) was incubated with beads and the beads-lysate mixture was put on rotating shaker overnight at 4 °C. Beads were washed by PBS and then eluted by sample buffer. The outcome was then analyzed by Western blotting assay.
